# Digital Twins for Just-in-Time Adaptive Interventions (JITAIs): Framework for Optimizing and Continually Improving JITAIs

**DOI:** 10.2196/72830

**Published:** 2026-03-06

**Authors:** Asim H Gazi, Daiqi Gao, Susobhan Ghosh, Ziping Xu, Anna L Trella, Predrag Klasnja, Susan A Murphy

**Affiliations:** 1 School of Engineering and Applied Sciences, Harvard University, 29 Oxford Street, Cambridge, MA, 02138, United States; 2 School of Information, University of Michigan–Ann Arbor, Ann Arbor, MI, United States

**Keywords:** digital twin, just-in-time adaptive intervention, mobile health, digital health, reinforcement learning, artificial intelligence, machine learning, precision medicine, simulation, deployment, JITAI, mHealth, AI, RL

## Abstract

In the context of digital health, just-in-time adaptive interventions (JITAIs) are nascent precision medicine systems that can extend personalized health care support to everyday life. A challenge in designing JITAIs is that personalized support often involves sophisticated decision-making algorithms. These decision-making algorithms can require numerous nontrivial design decisions that must be made between successive JITAI deployments (eg, hyperparameter selection for an artificial intelligence algorithm). Making design decisions between deployments—rather than during deployment—ensures intervention fidelity and enhances the ability to replicate results. Yet, each deployment can be costly, precluding the use of A/B testing for every design decision. How should design decisions be made strategically between JITAI deployments? This paper introduces “digital twins for just-in-time adaptive interventions (JITAI-Twins)” to address this question. JITAI-Twins are “digital twins of a subpopulation” (term used in the 2023 National Academies workshop proceedings on digital twins). JITAI-Twins are used to virtually simulate the potential outcomes of a JITAI’s design decisions for an upcoming deployment. Based on simulation results, design decisions are made for the deployed JITAI. To continually improve the JITAI, data collected during deployment are used to update the JITAI-Twin—and this bidirectional feedback between deployments and simulation environments continues. JITAI-Twins are thus “fit-for-purpose” (term used in the National Academies 2024 consensus report on digital twins) instantiations of the digital twin concept. In this paper, we elucidate the specifics and design process of JITAI-Twins, with examples of prior use in clinical settings. JITAI-Twins highlight continuity over the course of a JITAI’s optimization and continual improvement, emphasizing the need for bidirectional feedback between versions of a simulation environment and a JITAI’s deployments.

## Introduction

### Motivation

Just-in-time adaptive interventions (JITAIs) are promising intervention systems that have the potential to impact mobile health (mHealth) [1,2]. By leveraging wireless technologies such as wearable sensors to infer when an individual is at risk for negative change and/or receptive to positive change [3], JITAIs can proactively provide support in everyday life that is adapted to an individual’s health state or environmental context via “decision rules” [4]. Decision rules specify how intervention delivery should be adapted depending on “tailoring variables” such as the weather outside or an individual’s heart rate.

While adaptive intervention delivery can improve outcomes [5], it is nontrivial to design effective decision rules, and their design has increasingly relied on sophisticated artificial intelligence (AI) and control engineering methods. These methods optimize decision rules to better match the target individual or sample of individuals, rather than individuals from whom previous data were collected [6,7]. To use AI and control engineering methods, nontrivial design decisions must often be made, including which tailoring variables to use, which AI or control engineering method to use, or what hyperparameters should be selected for an AI algorithm [8].

Two competing forces make it challenging to make such design decisions for a JITAI. First, deployments can be costly and are critical to a JITAI’s continued development, especially in clinical settings [9,10]. It is imperative that a JITAI performs as optimally as possible when deployed. Second, JITAIs must remain autonomous during each deployment [7]. It is unscalable to require manual modifications to a JITAI’s decision-making algorithms during deployment, and unplanned modifications can compromise intervention fidelity and replicability [11,12]. The desire for a JITAI to perform optimally must be balanced with the need for a JITAI to operate autonomously—motivating the development of methods to evaluate and make informed design decisions before a JITAI is deployed.

### Digital Twins for Just-in-Time Adaptive Interventions

**Figure 1.**
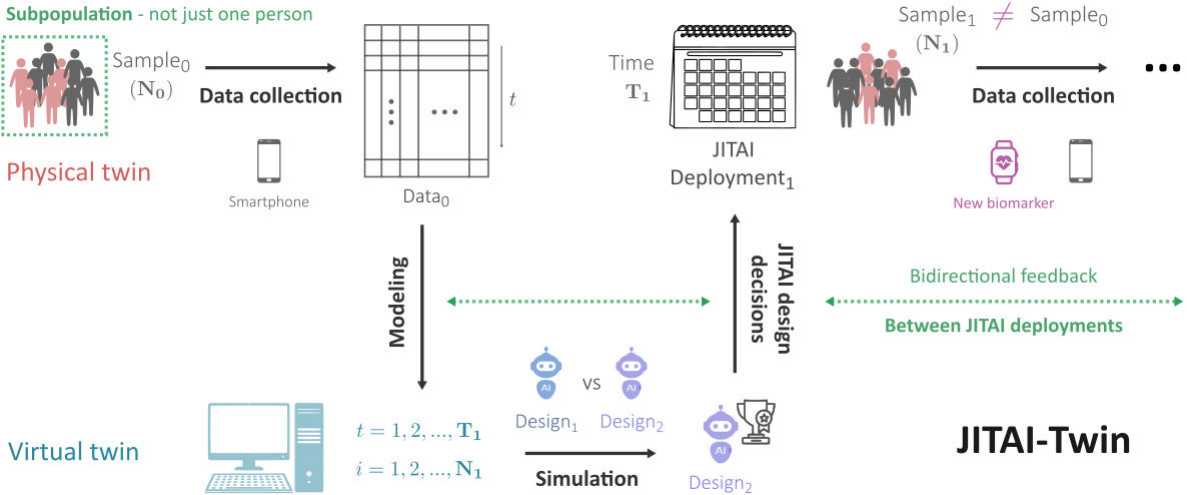
Digital twin for just-in-time adaptive intervention framework. Just-in-time adaptive interventions are designed for a subpopulation of individuals—not just a single individual, as highlighted in green to emphasize a key difference from most digital twins in health. This subpopulation serves as the physical twin. When a just-in-time adaptive intervention is deployed, data are collected from (a sample of) the physical twin. These data are used to construct simulation models that serve as the virtual twin. These models are used to simulate the performance of candidate decision-making algorithms for an upcoming deployment. The best-performing algorithm is then deployed within the just-in-time adaptive intervention. Data collected during deployment are then used to update the simulation models—and the modeling, simulation, decision-making, and data collection cycle continues—with bidirectional feedback taking place between just-in-time adaptive intervention deployments, as highlighted in green. Note the existence of data-deployment mismatches highlighted in pink, where data-deployment mismatches could involve differences in the prior data’s sample from the upcoming deployment’s sample or an inclusion of new variables. JITAI-Twin: digital twin for just-in-time adaptive intervention.

In this paper, we introduce the framework “digital twin for just-in-time adaptive intervention (JITAI-Twin)” and elucidate how the JITAI-Twin framework can be used to strategically make design decisions prior to a JITAI’s deployments (Figure 1). As discussed in a recent Consensus Report from the National Academies [13], digital twins are characterized by two components: (1) the “modeling and simulation to create a virtual representation of a physical counterpart” and (2) a “bidirectional interaction between the virtual and the physical.” This bidirectional interaction refers to the use of the virtual twin to inform decisions made for the physical twin, and the use of data from the physical twin to inform the virtual twin. JITAI-Twins include both virtual representation and bidirectional feedback components. JITAI-Twins take data from a JITAI’s target subpopulation (physical twin) and construct a set of virtual approximations to the subpopulation’s behavior (virtual twin). These virtual approximations are computational models of the subpopulation’s behavior when the JITAI is deployed (also known as “simulation environments”). Computational models are fit using prior deployment data and then used to simulate the subpopulation’s behavior. Simulations help evaluate multiple sets of potential design decisions (ie, candidate decision-making algorithms), to evaluate which candidate decision-making algorithms are likely to perform optimally. Based on simulation results, the JITAI design team can decide which decision-making algorithm to deploy on the JITAI for a sample of the subpopulation. This deployment produces additional data from the JITAI’s target subpopulation, and these data are again used to update and fit the computational models to simulate upcoming deployments and evaluate design decisions. This bidirectional feedback cycle continues for the lifespan of the JITAI-Twin.

JITAI-Twins are “fit for [the] purpose” of modeling a deployment subpopulation’s behavior over the course of a JITAI’s continual improvement [13-15], and this presents unique challenges that differ from challenges discussed in previous literature on digital twins for health [16-23] (refer to Section S1 of Multimedia Appendix 1 for a comprehensive overview of related work). A specific deployment’s sample rarely shares participants with a previous or follow-up deployment’s samples (Figure 1). Thus, models of the subpopulation are not fit or updated to replicate the behavior of specific individuals. Instead, the models should approximate the behavior and heterogeneity of the entire subpopulation, while only sampling from that subpopulation. In contrast, most digital twin frameworks in health focus on modeling a single individual to make model-informed decisions (eg, model predictive control for an artificial pancreas’ insulin delivery), where the person-specific model is updated with data specifically from the target individual [20]. Exceptions to this include “digital twins of a subpopulation” for rare disease treatment, discussed in the context of in silico clinical trials to better understand the potency of a drug [24]. Although only briefly mentioned in workshop proceedings of the National Academies without specific guidance on design or implementation examples [24], digital twins of a subpopulation for rare disease treatment are the most relevant example of digital twins. In fact, JITAI-Twins are another instantiation of the digital twin of a subpopulation concept. Other challenges that commonly arise for JITAI-Twins include “data-deployment mismatches.” Data-deployment mismatches are mismatches between the data available to construct the virtual twin and the upcoming real-world deployment characteristics that need to be simulated. Bidirectional feedback for JITAI-Twins takes place at the timescale of deployments, rather than any fixed unit of time (Figure 1). Due to the varying time lengths of deployments and the months or years that could pass between subsequent JITAI deployments, mismatches between previously collected data and future deployments can arise as society, science, and digital health technology evolve.

### This Paper’s Contributions

Compared to existing literature, the JITAI-Twin framework is the first instantiation of the digital twin of a subpopulation concept with associated guidance on design and implementation examples. Digital twins of a subpopulation are important to making informed design decisions and maintaining intervention fidelity when deploying a JITAI for a group of individuals. Relative to the literature on simulation testbed design for reinforcement learning (RL) algorithms for JITAIs [7], the JITAI-Twin framework highlights the need for bidirectional feedback. Feedback from virtual to physical involves design decisions that are informed via JITAI-Twin simulations. Feedback from physical to virtual helps improve a JITAI-Twin and ensure it matches the ever-changing deployment subpopulation for a JITAI. JITAIs are not designed and deployed as one-time efforts. JITAIs require continual improvement (eg, redesign and deployment) as scientific progress is made, technology evolves, and human behavior changes. Similarly, modeling, simulation, decision-making, deployment, and data collection should be viewed as an ongoing cycle (Figure 1).

This paper is organized as follows. We first provide a motivating example for JITAI-Twins based on a JITAI in the physical activity space that has required continual improvement over multiple deployments [5,25,26]. We then provide guidance on JITAI-Twin design and elucidate the step-by-step process of designing and updating JITAI-Twins. We also enumerate potential solutions to data-deployment mismatches that often complicate a JITAI-Twin’s design and updates. Penultimately, we discuss the iterative evaluation process of JITAI-Twins under the digital twin framework of verification, validation, and uncertainty quantification (VVUQ). Finally, we conclude with guidance on selecting a JITAI’s decision-making algorithm based on JITAI-Twin simulation results and discussion. We thereby make the following contributions:

1. Formalize the JITAI-Twin framework to instantiate digital twins of a subpopulation for the optimization and continual improvement of JITAIs.
2. Provide guiding principles for the design and updates of a JITAI-Twin.
3. Elucidate the design and iterative evaluation of JITAI-Twins, with examples of imputation and sensitivity analyses to address data-deployment mismatches.

## Motivating Example of a JITAI’s Continual Improvement: HeartSteps

HeartSteps is a JITAI that has been designed, optimized, and continually improved over the past decade to promote physical activity for sedentary adults [5,27]. In this section, we outline the development journey of HeartSteps, highlighting specific aspects of HeartSteps’ development that benefited from the use of a JITAI-Twin. We also highlight the challenges faced in designing the JITAI-Twin due to real-world considerations with HeartSteps.

The initial version of HeartSteps (“HeartSteps V1”) was aimed at increasing physical activity levels for sedentary adults by encouraging bouts of physical activity during daily life [5]. HeartSteps V1 consisted of a Jawbone Up wrist-worn activity tracker and a smartphone app that provided self-monitoring and “push” intervention components. The push interventions included 2 types of contextually tailored activity suggestions—intended to help individuals disrupt their sedentary behavior and take short (~10 minutes) walks throughout the day, respectively—and planning prompts, intended to help participants plan a longer activity bout for the following day.

To quantify the effects of the activity suggestions on subsequent physical activity and optimize future development of a JITAI to intelligently deliver activity suggestions targeting walking bouts (ie, walking suggestions), a 6-week microrandomized trial (MRT) was conducted. MRTs are designed to quantify how potential intervention components affect near-term outcomes of interest (eg, step counts), how these effects are moderated by context (eg, weather outside), and how these effects change over time [14]. The data from this initial MRT were then used to optimize the provision of both types of activity suggestions in the revised version of HeartSteps (“HeartSteps V2”).

To optimize the provision of walking suggestions for HeartSteps V2, the team developed an RL algorithm that personalized the provision of walking suggestions. This personalization was aimed at maximizing the impact of walking suggestions on the intended proximal outcome: step count in the subsequent 30 minutes [26]. To develop an appropriate RL algorithm and select its associated hyperparameters, the team designed simulation environments that we frame here as the first iteration of a JITAI-Twin for HeartSteps. The JITAI-Twin helped evaluate candidate RL algorithms and strategically select the appropriate RL algorithm to deploy [26].

A challenge in designing the JITAI-Twin for HeartSteps was the existence of data-deployment mismatches. First, HeartSteps V2 focused on a different population—individuals with type 1 hypertension living in a different geographical location (Seattle for V2 vs Michigan for V1)—and involved changes to the data collection methodology compared to HeartSteps V2. Second, the Jawbone sensor was no longer available, so HeartSteps V2 used Fitbit Versa smartwatches to track and collect physical activity data. Third, HeartSteps V2 included daily and weekly collection of ecological momentary assessment data that were different from the data collected when HeartSteps V1 was deployed, in part due to the team’s growing understanding of the psychosocial variables that mediate and moderate physical activity patterns. For example, an app engagement variable was included in HeartSteps V2 to help the RL algorithm personalize the provision of walking suggestions, but HeartSteps V1 did not include measurements of app engagement. After addressing these data-deployment mismatches (discussed in the section “Designing and Updating JITAI-Twins”), the JITAI-Twin simulation results informed the RL algorithm that was then deployed and evaluated for HeartSteps V2 [26].

To address limitations of the prior RL algorithm and incorporate the latest findings from the science of behavior change literature, the team is now working on an improved RL algorithm for deployment (this latest iteration of the JITAI is termed “HeartSteps V3” in this paper) [25,28]. The RL algorithm for HeartSteps V2 is optimized for increased step counts [26], but recent evidence demonstrates that long-term commitment to behavior change, such as the maintenance of physical activity, is more closely determined by affective attitudes toward the desired behavior, rather than short-term bouts of the desired behavior [29-31]. To incorporate this insight into HeartSteps V3, the upcoming deployment will use affective attitudes toward physical activity as outcome variables, and the walking suggestion component was redesigned to pair suggestions for walking with affectively positive stimuli to both increase receptivity to the suggestions and impact affective attitudes. The JITAI-Twin for HeartSteps is being updated accordingly based on the data collected during the deployment of HeartSteps V2. The JITAI-Twin is again being used to evaluate and select between candidate RL algorithms for HeartSteps V3 [25], and is again faced with data-deployment mismatches, as we describe in the section “Designing and Updating JITAI-Twins.”

## Guiding Principles to Follow for JITAI-Twin Design

When designing and updating a JITAI-Twin, several modeling and simulation decisions must be made. To inform these decisions, we propose the following guiding principles (Table 1) based on lessons learned from prior JITAI-Twin design and related work [7,26,32,33].

**Table 1.** Guiding principles for JITAI-Twin^a^ design.

Guiding principles	Brief description
Documentation	Document the purpose and plans for the design of a JITAI-Twin, adjustments made during design and updates, and simulation outcomes prior to each deployment
Team science	Include domain scientist(s), data scientist(s), and a deployment lead to ensure the necessary interdisciplinary expertise exists on the JITAI-Twin design team
Model generalizability	Use data modeling strategies that maximize generalizability from prior data to upcoming deployments
The devil is in the details	Judiciously investigate each aspect of a JITAI-Twin’s design, especially aspects that are critical to simulation performance
Refrain from data removal	To the extent possible, refrain from removing data that is representative of real-world deployments (eg, disengaged participants) when designing and updating the virtual twin

a JITAI-Twin: digital twin for just-in-time adaptive intervention.

### Documentation

A JITAI-Twin should be fit for purpose [13], and this purpose should be clearly documented. The design and update plans should be detailed, assumptions made in modeling the physical twin should be documented, and the objectives for each of the simulations conducted should be prespecified. During the JITAI-Twin design, adjustments to the plan will be necessary—these design decisions should be documented for computational reproducibility and continuity [34]. In general, a well-designed and documented JITAI-Twin has the potential to outlive its initial designers and provide a quality return on investment. Since JITAI-Twin simulations are conducted offline between JITAI deployments, computational cost is less likely to be a limiting factor compared to the time and effort in designing and maintaining a JITAI-Twin (ie, labor costs). Thus, like any computational resource, poor documentation can lead to wasted time and effort during updates to a JITAI-Twin, which can incur significantly more costs compared to spending time initially and at each update step on documentation.

### Team Science

Just as an interdisciplinary team is recommended when designing and deploying a JITAI, an interdisciplinary team is recommended when designing and leveraging a JITAI-Twin for decision-making [35]. The JITAI-Twin design team should, at a minimum, consist of the deployment lead, a domain scientist, and a computational scientist. The deployment lead is the researcher in charge of executing the JITAI deployment. The deployment lead’s insight is necessary to faithfully model practical aspects of a deployment and the target subpopulation (eg, expected disengagement from the JITAI). The domain scientist is the expert in physiology, psychology, etc, relevant to the health application (eg, suicidologist for a suicide prevention study). This domain scientist’s insight is necessary to design simulation models that reasonably approximate psychophysical changes and outcomes. Finally, the data scientist (eg, computer scientist, engineer, or statistician) is needed to mathematically abstract psychophysical characteristics into virtual constructs (eg, simulation models). Ideally, this data scientist has interdisciplinary training to improve cross-disciplinary communication and abstraction.

### Model Generalizability

“All models are wrong,” but “useful” models have explanatory power, capturing relationships that generalize from past data to future deployments [36]. We highlight 2 interpretations of explanatory power relevant to JITAI-Twin design [37,38]. First, explanatory power reflects a model’s ability to quantitatively capture known qualitative relationships, a characteristic often attributed to mechanistic models. This is especially important in the context of counterfactual outcomes, where a simulation model will need to generate plausible outcomes for alternative intervention options that were not selected in prior data. A recommended approach is to assess the limitations of prior data and ensure aspects of the model informed by domain knowledge account for blind spots in the data. For example, if prior data do not account for a portion of the JITAI’s target subpopulation, construct variants of the computational models that account for potential differences in behavior informed by domain knowledge [39]. Second, explanatory power refers to a model’s ability to generate data that align with real-world observations, a strength of data-driven machine learning models. JITAI-Twin models should balance the strengths of mechanistic and data-driven modeling to enhance generalizability [40]. Generalizability refers to how well computational models can explain or reconstruct data that were not used to train the models and has been extensively discussed in the literature [41,42]. A wealth of literature exists on the challenges of model selection for generalizability and approaches to consider [43,44]. In general, approaches to enhance generalizability ensure that a model’s complexity is commensurate with data availability. This is related to the bias-variance trade-off that has been shown empirically to be of value to model generalizability [45]. The bias-variance trade-off generally indicates that generalization error will follow a U-like curve, where large errors with too few parameters indicate too much bias and too little variance (ie, too little flexibility to capture the underlying patterns), and large errors with too many parameters indicate too little bias and too much variance (ie, too much flexibility causing the model to overfit). Recent work on the surprising performance of overparameterized machine learning models adds a new layer to this guidance, showing that the “double descent phenomenon” can be observed with overparameterized models (eg, deep neural networks) [46]. The complexity of model selection makes team science even more critical, as data scientists may have helpful intuition from experience that contradicts general guidance for a specific application setting.

Note that a JITAI-Twin’s simulation models should not only capture the underlying relationships of interest in the data (eg, relationships between intervention options and outcomes of interest), but also the unstructured variability within the data (ie, the “noise” characteristics). When candidate decision-making algorithms are evaluated, simulations will need to be conducted with stochastically generated noise to evaluate how well decision-making algorithms perform in settings that are not accounted for by the learned aspects of the models. Generalizable models are critical because a JITAI-Twin’s simulations inform real-world decisions for settings that will likely differ from prior data.

### The Devil Is in the Details

“Inattention to detail is the hallmark of mediocrity” for JITAI-Twin design [47]. JITAIs and real-world deployments are sophisticated, and because JITAI-Twins are used to make design decisions for real-world JITAI deployments, details cannot be overlooked or the risk of failure can become significant (eg, the Mars Orbiter that crashed partly due to a mismatch between metric and imperial units [48]). JITAI-Twin design must match the level of sophistication by attending to details such as the assumptions and limitations associated with a modeling approach (eg, assuming linearity vs modeling nonlinearities that may require more data). Of course, “perfect is the enemy of good,” so a JITAI-Twin design team must judiciously worry about aspects of a JITAI-Twin’s design that are critical to modeling the physical twin accurately (ie, “worrying selectively”) [36]. Team science helps guide the team in worrying selectively. Protocol details that are critical to model accurately can be highlighted by deployment leads. Domain scientists can prioritize which psychophysical relationships are most important to model. Data scientists can clearly articulate the limitations and the assumptions of modeling approaches they use. Due to the level of detail required in designing a JITAI-Twin, its design should be viewed as a research project itself. Adequate time must be budgeted for collaborative prioritization, modeling, simulation, evaluation, and iteration. Iteration will help data scientists learn what aspects of the target subpopulation and deployment are most important to model accurately for informative simulations. Iteration will also help deployment leads and domain scientists learn what correspondence is reasonable between the physical twin and virtual twin and maintain that standard.

### Refrain From Data Removal

Data removal should be avoided to the extent possible when designing a JITAI-Twin. JITAI-Twins approximate the true behavior of a target subpopulation using a JITAI. A fundamental truth of JITAI deployments is the harsh reality of user disengagement, missing data, etc. When disengaged users or users with insufficient data are removed or ignored, the JITAI-Twin becomes biased toward the subsample of prior participants or users that remained engaged [49,50]. However, future JITAI deployments will not only include engaged users, but will also include users who disengage. For a JITAI that seeks to personalize intervention delivery, disengaged users may in fact be the most important to consider [32]. Removing incomplete data would overlook users who may benefit the most from a well-designed decision-making algorithm. That said, if data are incorrect or are unrepresentative of the physical twin due to measurement or recording discrepancies, these data may need to be removed. Hence, the suggestion is not to avoid data removal entirely. Rather, the prudent JITAI-Twin designer should judiciously refrain from removing data representative of upcoming deployments.

## Designing and Updating JITAI-Twins

**Figure 2.**
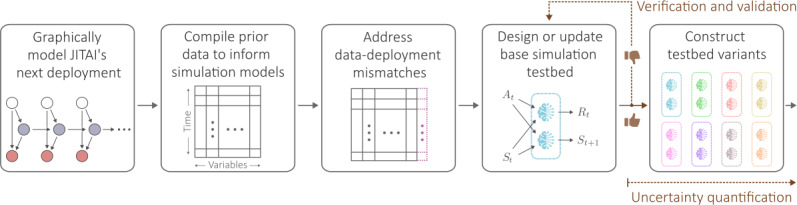
Overarching steps to designing (and updating) digital twins for just-in-time adaptive intervention. The 5 steps are illustrated from left to right and detailed in the main text. Verification and validation are iterative and continue until the base simulation testbed meets the desired performance. The construction of testbed variants is a form of uncertainty quantification that, along with other uncertainty quantification strategies, ensures just-in-time adaptive intervention design decisions are made in an uncertainty-informed manner. JITAI: just-in-time adaptive intervention.

Designing and updating a JITAI-Twin involves 5 overarching steps, as visually summarized in Figure 2:

1. The first step is to graphically model the upcoming deployment’s characteristics (eg, how often intervention can be delivered), intervention targets (eg, intervention will try to enhance positive affect toward physical activity), and target subpopulation (eg, sedentary adults). A recommended approach is to construct a causal directed acyclic graph (DAG) for the upcoming deployment to qualitatively represent expected user behavior in response to interventions, moderated by context variables [51,52].
2. The second step is to compile prior data that mirror the data to be collected for the upcoming deployment to the greatest extent possible. These data might be compiled from a previously conducted MRT, as was the case for HeartSteps V1 data [5,26]. Note that data from an MRT are not required, as will be discussed in the context of data-deployment mismatches.
3. The third step is to address data-deployment mismatches. For example, if the upcoming deployment involves an intervention component that was not present in prior deployments, the effect size for the intervention component must be imputed. The imputed effect size captures what domain scientists anticipate regarding the strength of the intervention component’s effects on outcomes, relative to non–intervention-related changes in outcomes (ie, the “signal-to-noise” ratio). The imputed effect size enables simulations of hypothetical responses to delivery of the intervention [53].
4. The fourth step is to construct a base “simulation testbed” informed by the graphical model, using the data compiled and imputation decisions made. A simulation testbed is defined as a collection of computational models used to simulate the performance of a JITAI’s decision-making algorithms. The base simulation testbed will include a set of simulation models (eg, outcome and state transition models) and parameters to vary. Some parameters will correspond to values imputed or specified to address data-deployment mismatches. Since JITAI-Twins are digital twins of a subpopulation and not of a single individual, the simulation models must capture heterogeneity between individuals in the subpopulation. The base testbed will need to be iteratively evaluated and redesigned until satisfactory evaluation results, as illustrated in Figure 2 and discussed in the next section, “Iterative Evaluation: VVUQ.”
5. The fifth step is to create variants of the base simulation testbed. These variants are used to evaluate how robust candidate algorithms are to potentially incorrect modeling assumptions. For example, if an intervention’s effect size had to be imputed (in step 3) or the intervention’s effects may differ from prior data, one can form testbed variants by varying the intervention’s effect size to understand how candidate algorithms perform across potential scenarios. This is a form of uncertainty quantification (Figure 2), as discussed in the next section, “Iterative Evaluation: VVUQ.” The variants and the base simulation testbed together form the virtual twin of the JITAI-Twin.

To make explanations more concrete, the remainder of this paper will focus on JITAI-Twins that simulate the performance of candidate RL algorithms (eg, the HeartSteps JITAI-Twin). The steps we describe remain applicable to simulating the performance of other decision-making algorithms for JITAIs, such as control engineering methods, decision trees, expert systems, etc [54]. RL algorithms are AI algorithms that iteratively optimize the selection of actions based on previously observed rewards and states [55]. In the context of JITAIs, RL algorithms are decision-making algorithms that can select between intervention options (actions) to optimize proximal outcomes (rewards) based on an individual’s tailoring variables (states). Example actions could include whether a physical activity suggestion is delivered or which suggestion is delivered. An example reward variable could include an individual’s step count as a surrogate for moderate-to-vigorous physical activity. Example state variables could include an individual’s environmental context, such as the weather or information about the individual, such as their current location. State-of-the-art JITAIs use RL algorithms to personalize intervention delivery, progressively determining which intervention option is likely to optimize outcomes based on the inferred state of an individual [26]. For a primer on RL and simulation modeling, refer to Section S2 of Multimedia Appendix 1.

### Graphically Model the Subpopulation and JITAI Deployment Characteristics

A graphical model helps ground subsequent JITAI-Twin design and update steps. With a graphical model, the research team can identify variables of interest and compile data accordingly for training models. The team can also identify which variables or relationships are important for which no data constitutes data impoverishment. When designing the base simulation testbed, the computational models used can then be structured according to the relationships specified in the graphical model. The graphical model can also help inform which variants to construct to investigate uncertainties in the graphical model.

**Figure 3.**
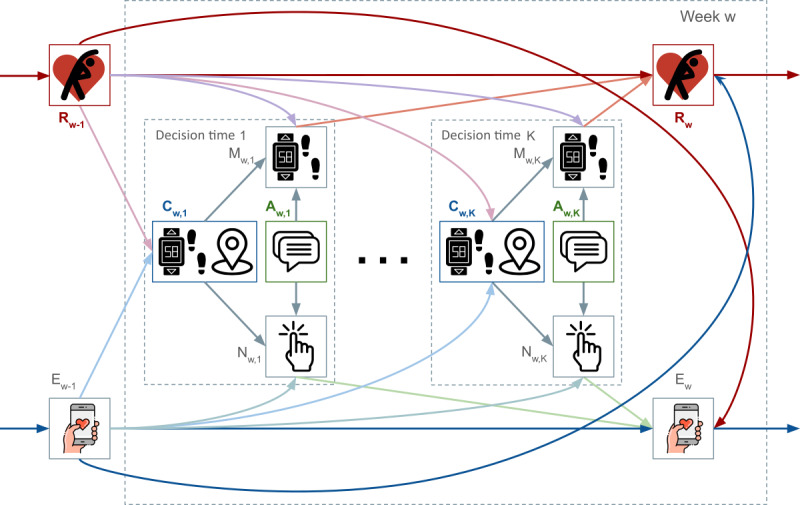
Example causal directed acyclic graph used for the HeartSteps digital twin for just-in-time adaptive intervention. The directed acyclic graph shown is being iteratively refined to graphically model HeartSteps V3. Each arrow indicates a causal relationship from the variable at the start of the arrow to the variable at the end of the arrow. The subscript w represents week (ie, w-1 refers to previous week), so the subscript w,1 refers to the first decision time within the week, while subscript w,K refers to the Kth decision time within the week. The context, C, represents an individual’s recent step count, GPS location, etc The proximal outcome, M, is the step count after an intervention is or is not delivered. Intervention, A, represents the decision to send a physical activity suggestion or not. Proximal outcome, N, represents whether a user clicks on an intervention message or opens the app. Intermediate outcome, E, represents a higher-level “app engagement” variable. The outcome variable, R, represents affective attitude toward physical activity.

For HeartSteps V3, the deployment characteristics and subpopulation behavior are currently being modeled by the causal DAG shown in Figure 3, adapted from Gao et al [25]. Note that this DAG is still under refinement but has undergone multiple iterations to collaboratively develop. A DAG visually describes causal relationships between the specified variables using directed arrows that are acyclic [51]. Importantly, arrows between variables that are omitted from the DAG reflect effects that are believed to be weak or nonexistent. Constructing a graphical model of this form requires the team to first identify the variables of importance to the upcoming trial. At a minimum, this consists of context variables (C in Figure 3), intervention options (A in Figure 3), and outcome variables (R and E in Figure 3). Identifying these variables translates to specifying states, actions, and rewards for the RL algorithm simulation testbed. The conceptual model should also specify mediators (M and N in Figure 3) that may affect the relationships between context, interventions, and outcomes. Since JITAI deployments involve decisions that are made over multiple time points (ie, sequential decision-making), it is important that the conceptual model captures time dependencies. For example, Figure 3 also illustrates dependencies across consecutive weeks and across consecutive days within a week. Assumed independence in time is also captured with the absence of arrows. Although independence between variables may not perfectly mirror the physical twin, it is important to selectively worry about the relationships most consequential to the upcoming deployment. A recommended approach to do this is to rank order the relationships between variables and decide on a cutoff point below which all relationships will be assumed inconsequential. Variants of the simulation testbed can be used to test the robustness of candidate decision-making algorithms to relationships that fall slightly below the cutoff point [25], as discussed in the later section “Construct Variants of the Base Simulation Testbed.”

Note that the variables included in the graphical model map to the subpopulation and deployment characteristics and not to the JITAI directly. This distinction is critical between JITAI-Twin models and a JITAI’s models used for decision-making. The JITAI may not measure or have access to all the variables that are important for simulations. The variables important for simulations are the variables needed to satisfactorily approximate how the target subpopulation will respond to the JITAI for the upcoming deployment. The graphical model needs to encompass all variables that are crucial for the JITAI-Twin’s simulation models.

### Compile Prior Data to Inform Simulation Models

Prior data are necessary to train and otherwise inform the computational models used for a JITAI-Twin. If prior deployment data are not available (ie, the JITAI-Twin is being designed for the first time, rather than being updated), the study team should identify prior data most relevant to the upcoming deployment’s simulations (similar variables, timescales of measurement, etc). The graphical model discussed in the previous section (“Graphically Model the Subpopulation and JITAI Deployment Characteristics”) can help guide data compilation. Relevant prior data should help inform relationships between variables in the graphical model.

For HeartSteps, the initial JITAI-Twin was designed using data from the HeartSteps MRT (ie, HeartSteps V1). MRT data are ideal for JITAI-Twin creation because MRT data contain dynamic measurements of outcomes and context relevant to the intervention components being investigated—and the provision of interventions is microrandomized. The microrandomization of intervention provision provides data for use in quantifying the relationships between intervention options and outcomes, and how the relationships are moderated by context variables. The outcomes and context variables map to rewards and state variables that a simulation testbed can generate in response to an RL algorithm’s simulated actions (ie, intervention provision).

When compiling prior data, it is important to avoid data leakage [56]. The study team should structure prior data in a way that mirrors how data will be used to inform algorithms for deployment. For example, some RL algorithms, such as Thompson Sampling algorithms used by HeartSteps, enable the use of informative Bayesian priors or other hyperparameters [57]. If the plan is to use a single dataset for both simulation and to inform the Bayesian priors, then the data should be split into disjoint datasets for modeling and Bayesian prior construction or hyperparameter selection, respectively [58].

### Address Data-Deployment Mismatches

The data compiled (see previous section, “Compile Prior Data to Inform Simulation Models”) are likely missing variables of interest from the graphical model (see previous section, “Graphically Model the Subpopulation and JITAI Deployment Characteristics”) or are deficient in some way when it comes to informing relationships of interest for the simulation models. We term these deficiencies “data-deployment mismatches.” Data-deployment mismatches are a reality of JITAI-Twin design and updates. During the continual improvement process, research teams often want to incorporate the latest innovations in sensing and intervention technology [59]. The science of behavior change, psychophysiology, and other relevant domain sciences is also progressing rapidly [28,60-62]. This progress and rapidly evolving technology often add additional variables or dependencies to consider for the upcoming deployment that are absent from previous deployment data. This absence of variables in prior data necessary to simulate upcoming deployment decisions is termed “data impoverishment.” Other forms of data-deployment mismatches include domain shift and sampling bias. Deployments may take place years apart, where characteristics of the subpopulation or their behavior may change. The time of the year or the specific sample investigated for each deployment may differ from previous deployments.

To characterize the data impoverishment faced for JITAI-Twin’s design or updates, identify the missing variables and/or relationships in the graphical model that cannot be fit using the data compiled. This data impoverishment can be overcome through judicious imputation decisions and the variation of unknown model parameters. These variations are a form of sensitivity analysis, where the uncertainty in the model parameters is propagated through to the simulation results (eg, if parameter x falls below value a, then decision-making algorithm 1 performs better than algorithm 2). Team science is critical to plausibly overcoming data impoverishment. Domain scientists and trial leads help ensure imputation and sensitivity analyses are conducted realistically.

Identifying domain shift and sampling bias requires careful comparison of the prior data, the overall subpopulation, and the upcoming deployment’s characteristics. For many deployments, it is not possible to fully anticipate the upcoming sample’s characteristics. However, it is important to recognize any oversampling or undersampling of a particular participant group in prior data. Sampling bias may also occur with respect to time. Prior data collected in the winter for a physical activity study will differ from what is expected if deploying a physical activity JITAI in the summer. When sampling bias is less obvious to the eye, methods to detect sampling bias include statistical tests to compare sample demographics and other characteristics to mean values or proportions known to be representative of the subpopulation. Domain shifts can still take place even if sampling was done in a representative manner. Over time, the characteristics of the subpopulation can change (eg, expectations of technology can change rapidly, as with the recent proliferation of generative AI tools), and how technology or a JITAI is deployed can also change. The detection of significant domain shifts (ie, “distribution shifts” or “dataset shifts”) is an active area of research, with methods that involve combining dimensionality reduction (of features such as baseline survey responses) and statistical testing performing the best in practice [63]. To overcome these data-deployment mismatches, domain knowledge can be used to adjust parameters of the simulation models according to expected differences between the domain or sample present in prior data and the upcoming deployment. Parameters of the simulation model that capture variability in the subpopulation or data collection environment can also be enlarged. Performing simulations with enlarged variances can help account for sources of variability in the upcoming deployment that may not have been observed in prior data due to sampling bias or domain shift.

For HeartSteps V3, outcome variables are impoverished [25]. Progress in the science of behavior change indicates that optimizing for short-term bouts of a desired activity is less indicative of sustained behavior change compared to optimizing affective attitudes toward the desired activity [28], which applies to the maintenance of physical activity [64-67]. Affective attitudes toward physical activity were not collected in prior data. To address this data impoverishment, affective attitudes toward physical activity are now being generated when updating the JITAI-Twin to simulate the upcoming deployment. Specifically, a linear state-space model is fit using system identification to infer latent (ie, not measured) changes in affective attitudes toward physical activity from observed (ie, measured) bouts of unprompted and prompted physical activity [25]. The relationship between affective attitudes toward physical activity and bouts of unprompted physical activity was captured in the DAG (Figure 3). For more examples of how data impoverishment has been addressed in the past for state and action variables, see Section S3 of Multimedia Appendix 1.

### Design the Base Simulation Testbed

Designing the base simulation testbed is an iterative process. Computational models are designed to generate (1) outcomes and (2) state transitions each time a candidate decision-making algorithm takes an action. These models must account for heterogeneity between individuals in the subpopulation. The computational models are then iteratively evaluated to ensure plausible outputs are generated. A detailed discussion of iterative JITAI-Twin evaluation is provided in the next section, “Iterative Evaluation: VVUQ.” Note that the model fitting and evaluation procedure for simulation models for JITAI-Twins differs from the procedures used for prediction and statistical inference models typically discussed in the machine learning and statistics literature. A simulation model for a JITAI-Twin is used to evaluate candidate decision-making algorithms, so the simulation model should not only be able to capture the underlying relationships between decisions, context variables, and outcomes of interest—but the simulation model should also be able to model noise characteristics properly. Simulations will need to be conducted with stochastically generated noise to evaluate how well candidate decision-making algorithms perform in settings that are not accounted for by the learned aspects of the models.

#### Outcome Modeling

Outcome generation requires a model that maps the state of the simulation environment and the action an RL algorithm takes to some set of outcomes. These outcomes can then be used to form rewards for an RL algorithm. Note the subtle distinction between outcomes and rewards. Rewards are used by an RL algorithm for learning and can differ from one RL algorithm to the next. Outcomes are generated by the JITAI-Twin regardless of which RL algorithm is used. The RL algorithms may use those outcomes to form their own reward signals.

Outcomes can be modeled probabilistically, $y\left(s,\;a\right)=P\left(Y_{t}=y|S_{t}^{w}=s,\;A_{t}=a\right)$. The superscript *W* emphasizes that this is the “world state” (ie, the all-encompassing state of the simulation environment, rather than the limited-in-knowledge state of specific RL algorithms to be evaluated). See the RL primer in the supplement for further explanation (Section S2 of Multimedia Appendix 1). The outcome model will need to be trained on prior data, after addressing data-deployment mismatches (section “Address Data-Deployment Mismatches”). The model used for *y* (*s*, *a*) can be of several forms [68]. See the work on HeartSteps V2 and HeartSteps V3 for examples [25,26]. The guiding principles presented in this paper and the availability of data should inform the model class and the model fitting process used [69,70].

#### State Transition Modeling

A state transition model is of the form $W(s^{\prime },\;s,\;a)=P(S_{t+1}^{w}=s^{\prime }|S_{t}^{w}=s,\;A_{t}=a)$. Again, the superscript *W* emphasizes that this is the “world state.” See Section S2 of Multimedia Appendix 1 for further explanation. This model quantifies the probability of reaching state S′, given that the current state is *S* and the RL algorithm for the JITAI takes action *a*. The simulation environment’s state transition model dictates how the simulation environment’s state changes over time in response to actions taken by the simulated JITAI, regardless of which simulated JITAI is used. Our ongoing work for HeartSteps V3 learns a state transition model to continually improve the JITAI-Twin for HeartSteps [25]. Note that by concatenating the next state, $S_{t+1}^{w}$ and the outcome, *Y_t_*, into a single vector, a single probabilistic model conditioned on *S*_*t*_ and *A*_*t*_ can be used to produce a vector of next state and outcome variables. This approach is used by Gao et al [25] and captured by the DAG in Figure 3, where next states are not differentiated from outcomes.

A simpler approach taken for HeartSteps V2 was to view state generation from a model-based data augmentation perspective [26]. Models are used only for the outcome function and to address state impoverishments. The prior data’s state sequences are then used as is to generate state transitions. This preserves the dependence across time in state variables but makes the simplifying assumption that the actions of the candidate decision-making algorithms have minimal effects on the sequences of states observed in prior data (eg, states such as weather and location). This decision is based on the principle that digital therapeutics tend to have little immediate effect on autonomous state dynamics relative to residual noise (ie, implicit assumption of a simpler RL algorithm called a “contextual bandit”).

#### Modeling Heterogeneity Between Individuals in a Subpopulation

A JITAI-Twin’s outcome and state transition models must account for heterogeneity between individuals [71]. Several approaches exist for modeling heterogeneity between individuals in a subpopulation. One approach that has been used for the HeartSteps JITAI-Twin is the construction of several user-specific models to represent a virtual cohort of users [25,26]. By fitting a user-specific model to each user present in prior data, the variability across model parameters can be used to quantify the heterogeneity across individuals. The user-specific modeling approach is useful when variability lacks a clear distributional structure. However, the downside of the approach is that it assumes prior data are representative of the subpopulation. An alternative approach is to assume that the heterogeneity across individuals in the subpopulation exhibits some structure (eg, normal distribution) or can be stratified in some way (eg, categorizing users by some phenotype). Stratified modeling or mixed-effects modeling (eg, with random effects that are normally distributed) can be used to form a single model or a smaller subset of models to simulate the responses of a virtual cohort of users. An inherent trade-off that exists is that imposing structure may lead to underfitting, but assuming no structure risks overfitting to prior data. More flexible data generation approaches (eg, via artificial neural networks) that use proxy features to account for user identities or characteristics could be used to generate a virtual cohort [72]. The downside of more flexible data generation methods is that they typically require extensive data to produce realistic outputs.

#### Additional mHealth Considerations

A few additional aspects of mHealth interventions are important to model. Engagement with an intervention tends to decay over time [5,73]. This can translate to reduced effect sizes of the intervention (captured by the outcome model) over the course of a simulated trial, as well as participant dropout. As long as data on participants who dropped out were not removed from the prior trial’s data, the prior trial’s data can be used to inform the expected dropout rate over the course of a simulated trial. User burden in response to the intervention should also be considered. If an intervention is delivered too often, habituation may reduce the effect size of the intervention over time (captured by the state transition model’s dynamics). Engagement may also diminish over time.

### Construct Variants of the Base Simulation Testbed

Variants of the JITAI-Twin’s base simulation testbed can be used to evaluate the robustness of candidate algorithms to potentially incorrect assumptions made during graphical modeling or decisions made to overcome data-deployment mismatches. The use of testbed variants is critical because the base simulation testbed on its own will fail to replicate characteristics of the upcoming deployment. However, the base testbed along with a well-designed set of variants can be used to cast a broader net over the possible scenarios the JITAI may encounter in the upcoming deployment.

The variants constructed should all satisfy “matching conditions.” Matching conditions are defined here as marginal statistics or other outputs of the simulation testbeds that must be met to ensure scientifically plausible simulations. For example, if the study team decides that the degree of between-subject variability observed must be greater than some value, then plausible variants must produce between-subject variability above that value. Calibration can be used to ensure matching conditions are satisfied.

For HeartSteps V3, a simplified DAG is used to inform the RL algorithm (simplified version of the DAG shown in Figure 3; see Gao et al for details) [25]. Certain variants of the simulation testbed intentionally cause mismatches between the DAG used to inform the RL algorithm and the DAG used to generate the JITAI-Twin simulation testbed’s rewards. The RL algorithm selected for deployment should be robust to these mismatches. Imputed effect size trajectories were also varied to construct additional variants. These variants are used to test the performance of candidate RL algorithms for the entire range of plausible effect sizes (a matching condition) that could be observed during the upcoming deployment.

## Iterative Evaluation: VVUQ

**Figure 4.**
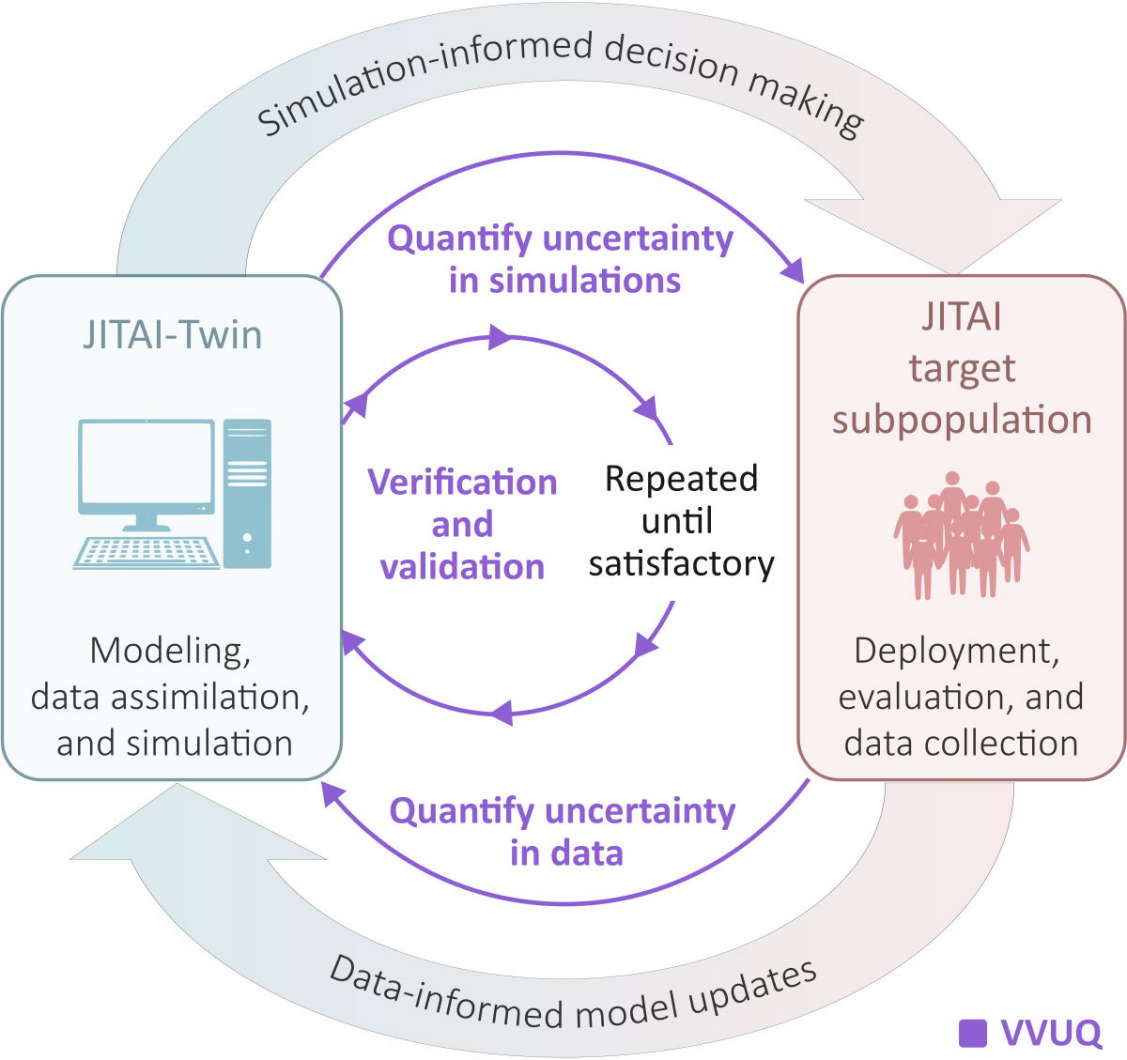
Verification, validation, and uncertainty quantification for digital twins for just-in-time adaptive interventions. Verification, validation, and uncertainty quantification is an iterative process. For each iteration of the digital twin for just-in-time adaptive intervention, multiple repetitions of verification and validation are often necessary before satisfactory simulation performance is achieved. Uncertainties must then be quantified in simulations to inform decision-making for a just-in-time adaptive intervention’s deployment. Data from a just-in-time adaptive intervention’s deployment will contain sources of error that also need to be quantified when updating the digital twin for just-in-time adaptive intervention’s computational models. The verification, validation, and uncertainty quantification cycle continues. JITAI: just-in-time adaptive intervention; JITAI-Twin: digital twin for just-in-time adaptive intervention; VVUQ: Verification, validation, and uncertainty quantification.

The evaluation process for JITAI-Twins is iterative and aligns with the digital twin evaluation framework of VVUQ [13,74], as shown in Figure 4. Verification ensures that the virtual constructs (eg, simulation models) created computationally (eg, using floating point approximations) mirror the desired mathematical descriptions. Validation ensures that the digital twin adequately represents the physical twin for the purposes of its design. Uncertainty quantification encompasses sensitivity analyses, the quantification of confidence in simulation results, and the quantification of uncertainty in data collected when designing and updating a JITAI-Twin. Sensitivity analyses determine how simulation outcomes are affected by varying learned parameters and/or modeled variables. Confidence measures are produced by performing numerous stochastic simulations and by using statistical methods to quantify confidence and credible intervals (ie, uncertainty quantification) for simulation outcomes. Data uncertainty can arise due to corrupted data, among other sources of uncertainty. Although difficult, characterizing sources of uncertainty (eg, heterogeneity in the subpopulation or stochasticity in simulation) and quantifying uncertainty accordingly are critical tasks.

### Verification

Verification is necessary for any computational model or piece of code. Standard practices to ensure proper code execution, negligible floating-point approximation errors, and appropriate data generation should all be first-pass evaluation criteria. One verification technique relevant to generative modeling is replication analysis. One can ensure replicable simulations by keeping track of the seeds used for random number generation and rerunning for verification. Other ways to improve verification include publicly sharing the code, procedural information, and data (if possible). Other researchers and practitioners can then participate in verification and improvements to the procedures and code.

### Validation

Data from the upcoming deployment are necessary to truly validate a JITAI-Twin’s simulations. However, once data are collected for the upcoming deployment, it is too late to update the JITAI-Twin according to validation results. Thus, alternative strategies for predeployment validation are necessary to ascertain JITAI-Twin quality. We separate JITAI-Twin validation strategies into 2 categories: mechanistic and empirical.

#### Mechanistic

Mechanistic validations are used to determine whether the generated simulation data are reasonable in the context of domain science and deployment experience. Specific examples include simulated engagement with a JITAI or participant dropout. Decaying engagement with an intervention and participant dropout rates should be modeled in a manner that aligns with deployment expectations. Other examples include simulated dynamics for state variables, outcomes, etc A concrete example from HeartSteps is to evaluate whether generated step count outcomes should decrease over time when the simulated virtual participant experiences an increase in positive affect toward physical activity (should generally not be the case in this example).

#### Empirical

Empirical validation requires detailed, data-driven evaluation of the JITAI-Twin’s generative models and their outputs (see the supplementary material of Gao et al for an example from HeartSteps) [25]. Empirical evaluation strategies can be separated into 3 categories. The first category involves goodness-of-fit assessments on prior data used to fit the simulation models. Goodness-of-fit measures describe how well the trained model can generate outputs (in the absence of noise or residual components) similar to the prior data outputs when given prior data inputs (eg, coefficient of determination for regression). Coefficients of the model can also be assessed to ensure that the weights are of the appropriate scale, sign, and magnitude according to domain science.

The second category of empirical validation involves out-of-sample prediction (eg, using held-out data). For models fit to several participants’ data simultaneously, a subset of participants’ data can be held out to evaluate agreement between simulated and real data when the models are used to predict the held-out outcomes and state transitions given the held-out states and actions. When simulations are conducted without noise, generated data should align with the held-out data, on average (ie, the expected values, in a statistical sense, should align). When simulations are conducted with noise (as is done over multiple iterations when evaluating decision-making algorithms), the out-of-sample data should align with the distribution of data stochastically generated by the models. Importantly, the aim of out-of-sample prediction is not to replicate the held-out data during data generation. The aim of out-of-sample prediction is to instead evaluate how well marginal statistics and other diagnostic metrics agree with held-out data. In cases where a set of participant-specific models are trained to act as virtual participant models, participant-specific data can be held out and used for validation.

The third category of empirical validation diagnoses whether the models generate data that satisfy diagnostic checks. This is related to model calibration [75,76]. These diagnostic checks evaluate whether statistics computed across multiple simulated trials (eg, effect sizes and variability across virtual participants [7,25]) agree with the statistics computed from physical trials. It is recommended to inspect several statistics when validating a JITAI-Twin’s generated data.

### Uncertainty Quantification

Uncertainty quantification ensures that decisions informed by a JITAI-Twin are made with an understanding of the uncertainty in JITAI-Twin modeling and simulation results [77]. In this context, uncertainty quantification refers to computing measures of variability and alternative outcomes to accompany a JITAI-Twin’s simulation results, such as 95% CIs for a candidate decision-making algorithm’s simulated performance or simulation performances using a different testbed variant. There are several sources of uncertainty that, to the best of the JITAI-Twin design team’s capacity, need to be considered, quantified, and propagated to quantify uncertainty for a JITAI-Twin’s simulation results. We outline several important sources of uncertainty for JITAI-Twins in this subsection.

One source of uncertainty is stochasticity in simulations. Stochasticity is critical because simulations often involve several probabilistic outcomes that may differ from one simulation to the next (eg, the outcome of a coin flip). Stochasticity manifests either through probabilistic modeling used for the outcome and state transition models or probabilistic decision-making via a candidate decision-making algorithm. To account for this stochasticity, several seeds should be used for random number generation to investigate the variability in results across multiple simulation runs. CIs can be constructed accordingly, and algorithm evaluation decisions can then be made with a notion of statistical significance [32]. Note that for implementation purposes, the simulations corresponding to each random seed can be parallelized to potentially save time.

An additional source of uncertainty is the uncertainty in trained parameters and imputed variables. Parameters of a simulation model that are trained using prior data may not be accurate in representing upcoming deployment characteristics due to limitations in the quality and quantity of prior data. Variables that are imputed using domain knowledge to address data impoverishment may behave differently from what is observed on average in domain sciences. Sensitivity analyses (eg, via variants of a simulation testbed) can be a useful way to investigate how simulation results vary as a function of changes in imputed variables or changes in learned parameters [78].

A final source of uncertainty we discuss here is uncertainty in the data compiled from prior deployments and studies, as shown in Figure 4. Accounting for uncertainty in collected data is important because the data compiled will significantly influence what the JITAI-Twin’s simulation models capture during training. Uncertainty in compiled data can exist due to inference and measurement errors. Inference errors exist when a computational model is used to map measured data to variables of interest, such as context or outcome variables (eg, an algorithm that computes step counts will produce estimates with error). Measurement errors exist due to the inherent uncertainty in measurements, such as surveys of psychological constructs or sensor-based estimates of quantities that can be corrupted by external sources of noise. Distinguishing uncertainty in measurements from uncertainty in model predictions due to limited knowledge has been discussed extensively in the statistics and machine learning literature [79]. These sources of uncertainty can be simulated and propagated through to the simulation results by introducing noise terms with variances that can be scaled in magnitude to help quantify the sensitivity of simulation results to these errors [80].

### VVUQ Is Continual

The VVUQ process is continual for a JITAI-Twin. Once a JITAI-Twin is used to inform decision-making in practice, a JITAI-Twin should be reevaluated following the completed deployment. This reevaluation will help inform the JITAI-Twin prior to its use in decision-making for the subsequent deployment—and the cycle continues, as depicted in Figure 4.

## Evaluating Candidate Algorithms According to JITAI-Twin Simulation Results

**Figure 5.**
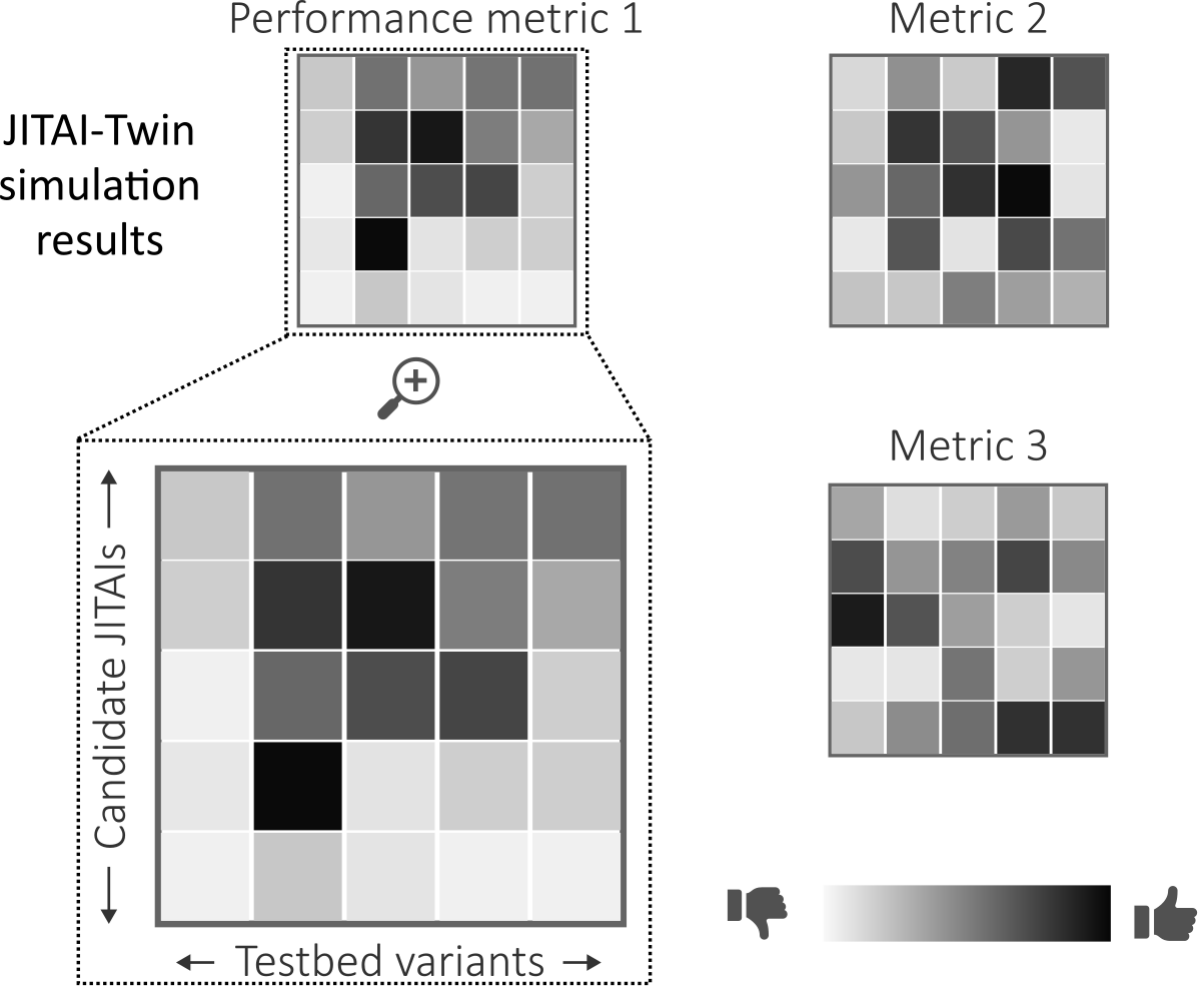
Illustrative example of a digital twin for just-in-time adaptive intervention’s simulation results. For each simulation testbed variant of a digital twin for just-in-time adaptive intervention, candidate digital twin for just-in-time adaptive intervention decision-making algorithms will be evaluated according to metrics that the team deems useful for deciding which algorithm to deploy onboard the real digital twin for just-in-time adaptive intervention. Performance metrics may not always agree with respect to which algorithm to select. Trade-offs must often be made based on the performance priorities for the digital twin for just-in-time adaptive intervention to be deployed. JITAI: digital twin for just-in-time adaptive intervention; JITAI-Twin: digital twin for just-in-time adaptive intervention.

Once a JITAI-Twin has been evaluated satisfactorily, the JITAI-Twin’s simulation results can be used to decide which decision-making algorithm to deploy as part of the upcoming JITAI. Note that these simulations should be conducted with noise variables that match the characteristics of the noise observed in previously collected data (eg, informed by the simulation models’ residuals when fit to prior data). This helps evaluate how well candidate decision-making algorithms will perform in real-world settings that are not completely accounted for by the learned aspects of the simulation models. For each candidate decision-making algorithm, the JITAI-Twin will produce a set of simulation results. These results will correspond to multiple simulated deployments for each variant of the base simulation testbed, as illustrated in Figure 5 (for a real example of simulation results, see Trella et al [7]). The goal at this stage is to identify the specific algorithm to be used for deployment based on the results shown across all simulations.

The metrics used to evaluate candidate algorithms and decide which to deploy will depend on the research team’s priorities. If the primary goal of an intervention is to elicit a positive impact on average across the entire deployment sample, then a measure of central tendency across all virtual participants is likely sufficient. An example measure would be the mean across all virtual participants of the average cumulative reward across all simulated deployments (ie, $\frac{1}{N}\sum_{n=1}^{N}\frac{1}{S}\sum_{s=1}^{S}\sum_{t=1}^{T}R_{t,\;s,\;n}$, where s indexes random seed, *n* indexes virtual participant, and *t* indexes time within each simulated deployment). However, if one goal of an intervention is to ensure that individuals with disorders that are more treatment resistant experience some benefit, then a lower percentile might be taken into consideration (eg, 25th percentile of $\frac{1}{S}\sum_{s=1}^{S}\sum_{t=1}^{T}R_{t,s,n}$). Other metrics could be used to investigate considerations related to time. For example, if an intervention trial may be terminated early if outcomes are not met [81], the algorithms may be evaluated for an initial subset of time (ie, instead of $\frac{1}{N}\sum_{n=1}^{N}\frac{1}{S}\sum_{s=1}^{S}\sum_{t=1}^{T}R_{t,s,n}$, one could use $\frac{1}{N}\sum_{n=1}^{N}\frac{1}{S}\sum_{s=1}^{S}\sum_{t=1}^{T^{`}}R_{t,s,n}$ for $T^{\prime }<T$). If early participant disengagement is a primary concern, several T` values can be used to compare the speed of learning across the candidate RL algorithms. Table 2 summarizes the 3 example metrics described. Note that if multiple metrics are used to compare algorithms, design trade-offs will be necessary, as illustrated in Figure 5.

**Table 2.** Example metrics to evaluate candidate algorithms^a^.

Example metric	Use case	Formula
Mean cumulative reward	Choose the algorithm that elicits the greatest positive impact on average across the entire deployment sample	$\frac{1}{N}\sum_{n=1}^{N}\frac{1}{S}\sum_{s=1}^{S}\sum_{t=1}^{T}R_{t,s,n}$
Lower percentile of the cumulative reward (eg, 25th percentile)	Choose the algorithm that improves outcomes the most for those that are least benefited (eg, treatment resistant)	Lower percentile of $\frac{1}{S}\sum_{s=1}^{S}\sum_{t=1}^{T}R_{t,s,n}$
Mean, early terminated reward	Choose the algorithm that most improves the mean positive impact within a shorter time period than the full deployment	$\frac{1}{N}\sum_{n=1}^{N}\frac{1}{S}\sum_{s=1}^{S}\sum_{t=1}^{T`}R_{t,s,n}$ for $T^{\prime }<T$

a *s*∈{1,2,...,*S*} indexes random seed, *n*∈{1,2,...,*N*} indexes virtual participant, and *t*∈{1,2,...,*T*} indexes time within each simulated deployment.

For HeartSteps V2, the average total reward was used as the primary metric [26]. For HeartSteps V3, the average and 95% CIs for the cumulative reward are also reported [25]. CIs provide an indication of variability across virtual participants.

## Discussion

### Implications for Research and Practice

The JITAI-Twin framework we elucidate and the guiding principles we provide are informed by prior work in designing RL algorithms for JITAIs. Aside from the HeartSteps JITAI described in Guiding Principles to Follow for JITAI-Twin Design [25,26], 2 RL-empowered JITAIs that have been deployed in clinical trials were used to inform this paper. For the “Oralytics” JITAI designed to improve oral health in disadvantaged populations [82], the first iteration of a JITAI-Twin was designed and used to inform design decisions (hyperparameters of the RL algorithm’s reward [53]) for the JITAI’s clinical trial that started in fall 2023 and concluded in summer 2024 [83]. A unique challenge faced when designing the Oralytics JITAI—that was simulated as part of the JITAI-Twin—was delayed access to oral self-care behavior data from participants due to the use of a commercial smartbrush. Issues with data accessibility are common with commercial products and should be simulated by a JITAI-Twin. For the “MiWaves” JITAI designed to reduce cannabis use in emerging adults, the first iteration of a JITAI-Twin was used to select the reBandit algorithm over baseline approaches [32]. MiWaves was then deployed in a preregistered feasibility and acceptability study which was completed in fall 2024 [84]. A challenge faced with MiWaves was that the reward for the RL algorithm was a measure of engagement with the JITAI, but engagement with a JITAI is latent and difficult to quantify. A surrogate reward was thus defined and used for JITAI-Twin simulations to evaluate candidate algorithms. In general, the JITAI-Twin framework can be extended to many other domains, so long as the guiding principles of team science and attention to detail are followed to address domain-specific challenges.

The more costly it is to evaluate design decisions in the real world, the more valuable a JITAI-Twin is for optimizing and continually improving a JITAI. A/B testing is a tried-and-true approach to comparing design choices in practice [85], and is the basis for MRTs and other optimization experiments [14]. A significant downside to A/B testing and optimization experiments is that factorial designs can quickly become untenable when the cost of collecting data is nonnegligible and several nonobvious design decisions need to be made prior to the deployment of a JITAI. Especially for JITAIs that leverage RL and control engineering approaches, several design decisions must often be made just for model selection [8,43]. On the other hand, some design decisions may be difficult to model using prior data and simulate via a JITAI-Twin. An example would be whether a new intervention component that differs substantially from previous components should be added to a JITAI (eg, noninvasive vagus nerve stimulation in tandem with mindfulness interventions for stress management [86]). Optimization experiments are best reserved for these difficult-to-simulate design decisions, especially if the experimental cost of evaluating decisions is non-negligible. Thus, the recommended path forward is to use JITAI-Twins and optimization experiments in tandem to optimize and continually improve JITAIs.

Compared to person-specific digital twins, there are practical advantages to using digital twins of a subpopulation when optimizing and continually improving a JITAI. A person-specific digital twin becomes obsolete as soon as the specific person is no longer being considered for upcoming deployments of a JITAI. Digital twins of a subpopulation remain useful so long as the JITAI is being continually improved for the target subpopulation. Practically speaking, digital twins of a subpopulation also reduce the need to collect large amounts of data from specific individuals. Although person-specific digital twins enhance personalization and circumvent the need to model subpopulation heterogeneity, prior work shows that months of exploratory data collection can be required to satisfactorily construct person-specific models for a JITAI [87]. In behavioral settings where adherence remains an issue and disengagement often spells the end of an individual’s participation, collecting large amounts of data from a single individual may be a challenge. Future work should consider balancing the 2 approaches to better balance learning within a deployment for improved personalization (person-specific) and learning between deployments to continually improve a JITAI system (subpopulation-level).

Other opportunities for future research on JITAI-Twins include the use of large language models to improve the abstraction of domain science (often described using natural language) into computational constructs, the use of large language models to simulate participant interactions with a JITAI [88], and the use of foundation models to leverage large sums of previously collected data that may be openly available but rife with data-deployment mismatches. See Section S4 of the Multimedia Appendix 1 for further discussion of future work.

### Limitations 

Our discussion of JITAI-Twins is limited in a few respects. The underlying concepts behind JITAI-Twins may find broader applicability beyond JITAI deployments. Other adaptive digital interventions may also benefit from digital twins. However, this paper is restricted to JITAI applications to ensure guidance is informed by practical experience. The example JITAI-Twins in this paper only include those that the coauthors of this paper have designed. Although others have designed JITAIs empowered by RL algorithms [89,90], the use of JITAI-Twins is not yet standardized and the publication of simulation environments and results is not yet commonplace, limiting our ability to include other simulations and JITAIs as examples. One of the goals of this paper is to bring JITAI-Twins to the forefront of discussion to overcome this gap. Future work is also necessary to better elucidate practical considerations, such as the computational and labor costs associated with designing and maintaining a JITAI-Twin and potential comparisons with the costs of conducting optimization experiments for the same design decisions. Other practical considerations include scalability. Although the framework as described is not dependent on the amount of data available or the scale of a JITAI’s deployment, future work is necessary to evaluate the generalizability of the JITAI-Twin framework to larger-scale deployments. Guidance is needed on adapting and improving RL algorithms based on JITAI-Twin simulation results. Although the previous section of this paper provides initial guidance on RL algorithm selection, we leave further discussion to future work, as the scope of this paper focuses on JITAI-Twins. Future work is also needed to empirically validate the benefit of each of the proposed guiding principles and design steps for JITAI-Twins to potentially improve or provide additional evidence for aspects of the framework.

### Conclusions

In this paper, we present the JITAI-Twin framework as an instantiation of “digital twins of a subpopulation” for JITAI optimization and continual improvement [24]. We detail how JITAI-Twin simulation results can be used to make difficult design decisions, such as which decision-making algorithm to deploy as part of a JITAI. This serves as information flow from the virtual twin to the physical twin. We also show how data collected from a JITAI’s deployment can be used to update the JITAI-Twin prior to the subsequent deployment, demonstrating information flow from the physical twin to the virtual twin. JITAI-Twins are thus “fit for [the] purpose” of simulating the behavior of a JITAI deployment’s subpopulation [13]. The impact that JITAI-Twins can have would be greatly enhanced through collaborative efforts to share computational resources for designing and evaluating JITAI-Twins (eg, codebases); curate data that could be used to initialize, train, or update JITAI-Twins; and establish benchmarks to help practitioners better understand when a JITAI-Twin is ready to inform decisions or to help researchers evaluate new methods for JITAI-Twin modeling or simulation. Toolboxes that enhance the accessibility of JITAI-Twin design (eg, as a possible addition to the JustIn platform [91]) would also facilitate more widespread adoption of JITAI-Twin methods to improve the optimization and continual improvement of JITAIs.

## Supplementary material

10.2196/72830 Multimedia Appendix 1 Includes a more extensive review of related literature, a primer on reinforcement learning algorithms and simulation models, additional examples of data impoverishment faced during digital twin for just-in-time adaptive intervention design and updates, and additional discussion on open questions and future directions in digital twin for just-in-time adaptive intervention design.

## Abbreviations

AI: artificial intelligence
DAG: directed acyclic graph
JITAI: just-in-time adaptive intervention
JITAI-Twin: digital twin for just-in-time adaptive intervention
mHealth: mobile health
MRT: microrandomized trial
RL: reinforcement learning
VVUQ: verification, validation, and uncertainty quantification
