# Supplementary material for: Digital Twins for Just-in-Time Adaptive Interventions (JITAIs): Framework for Optimizing and Continually Improving JITAIs
Source: J Med Internet Res. 2026 Mar 6;28:e72830. doi: 10.2196/72830 (PMC12978917; doi:10.2196/72830)
Supplement: Multimedia Appendix 1 [file jmir-v28-e72830-s001.docx]

**Supplementary Material**

**Digital Twins for Just-in-Time Adaptive Interventions (JITAI-Twins): A Framework for Optimizing and Continually Improving JITAIs**

Asim H. Gazi, PhD, Daiqi Gao, PhD, Susobhan Ghosh, Ziping Xu, PhD, Anna L. Trella, Predrag Klasnja, PhD, Susan A. Murphy, PhD

**S1. Related Literature**

The concept and original definitions of a digital twin date back decades [1], [2]. Modeling and simulation have served as cornerstones of data science, healthcare, and reinforcement learning (RL) [3], [4], [5]. In this section, we provide additional background on digital twins and dive into the work on digital twins for health thus far. We then bridge the gap between recent literature in the digital twins for health space and clinical trial simulation. We highlight recent work using clinical trial simulation to optimize model-informed precision dosing (MIPD) as being most relevant. Finally, we connect this MIPD research to RL, discuss simulation test beds in RL, and highlight Sim2Real as being the most relevant literature in AI. See the main text for a synthesis of related work and the specific aspects of JITAI-Twins that are novel relative to previous literature.

**Digital Twins**

Digital twins are sets of virtual information constructs (e.g., computational models) that approximate a real system or “system-of-systems.” The real system (e.g., participant) or system of systems (e.g., deployment subpopulation) is termed the *physical twin* [6], [7]. The virtual constructs are called the *virtual twin* or are simply referred to as the digital twin itself. The digital twin has some predictive capacity and is used to inform decisions that affect the physical twin. These decisions are made by modeling scenarios of interest and virtually simulating the effects of various decisions on the physical twin. The virtual constructs or models of the digital twin used for simulation are dynamically updated with data from the physical twin. This forms a bidirectional feedback loop between the digital and physical twins: the digital twin is used to inform decisions made for the physical twin, and data from the physical twin is used to update the digital twin [6]. This concept of a digital twin stems from the aerospace industry, where digital twins have been used to virtually mirror the lifecycle of a physical vehicle or system [8], [9]. Creating these digital twins enabled simulations of various conditions for the physical system – simulations that would be too risky or impossible to experimentally investigate.

The two components that characterize a digital twin of (1) modeling and simulation and (2) bidirectional feedback between the virtual and physical must be “fit for purpose” [6]. This means that the purpose of the digital twin should dictate the spatiotemporal resolution at which the models are fit, the frequency of bidirectional feedback, etc. For example, if the purpose of a digital twin is to approximate the millisecond-level effects of optogenetic stimulation on a specific neuron population [10], then modeling and simulation need to be performed, at a minimum, at the spatiotemporal resolution of the neuron population on the order of milliseconds. Modeling the system with less resolution would ignore behavior relevant to stimulation’s effects on the physical twin. For bidirectional feedback, however, the timescale can be quite different. If changes to the algorithm that autonomously decides what stimulation parameters to use (i.e., the “control algorithm”) can only be made on a daily basis, then bidirectional feedback does not need to take place faster than daily – even if the stimulation current (i.e., actual input to the physical system) could be changed every millisecond. This is because feedback from the virtual to the physical – changes to the control algorithm – can only take place every day. It would be superfluous to update the virtual twin more frequently using data from the neuron population (i.e., physical twin) because those updates would not help in changing the control algorithm (i.e., the decision that can be made to affect the physical twin). It is critical to understand this idea of “fit for purpose” when instantiating digital twins for particular applications.

In health settings, digital twins have thus far focused on modeling single individuals or their subsystems (e.g., organ systems or specific organs) [11], [12], [13], [14], [15], [16]. Exceptions to this include some work on digital twins for healthcare management [17], digital twins for public health [18], and digital twins for in-silico clinical trial design [19]. Digital twins for in-silico clinical trials are most similar to JITAI-Twins. Digital twins for in-silico clinical trials focus on modeling and simulating patient responses for enhanced causal inference. This is done by simulating outcomes that are not present within the actual data collected (i.e., counterfactuals). For example, digital twins are used to simulate how the same patient who received treatment would have responded in the control arm (e.g., RCT-Twin) for enhanced post-study analyses [20], [21]. Other examples of digital twins for clinical trial design more closely mirror clinical trial simulation, where the goal of the digital twin is to improve the trial’s design. More specifically, the digital twin enables simulations that help ensure statistical power and other trial considerations are met for the physical twin (e.g., Unlearn.AI) [22]. In contrast to these examples, JITAI-Twins are not designed to generalize post-study analyses or inform an experimental trial’s design. Instead, JITAI-Twins are used to inform the intervention itself (recall that the decision-making algorithm is part of the intervention) that is being investigated for an upcoming deployment. This is done by evaluating candidate decision-making algorithms for a proposed JITAI in simulation. Digital twins for in-silico trials are instead more related to clinical trial simulations.

**Clinical Trial Simulations**

Clinical trial simulations have been used for decades to inform the design of pharmaceutical trials [23]. Clinical trial simulations improve the likelihood that trial goals are met by informing decisions that need to be made regarding a trial’s design [24]. These decisions include study sample (e.g., study population), experimental design (e.g., length of a study), or even the dosing regimen for a pharmaceutical. A trial’s goals could include some desired statistical power, diversity in the analytical sample, or sufficient data on safety. For a clinical trial simulation, virtual constructs are developed to model an upcoming clinical trial. These constructs include models of how a pharmaceutical interacts with the human body (e.g., pharmacokinetics-pharmacodynamics models), models of participant recruitment and dropout, and other models of covariates relevant to the clinical trial at hand (e.g., relationships between participant characteristics to ensure the simulated samples are plausible). These models are used to simulate the pharmaceutical’s effects on a virtual sample, accounting for trial execution aspects such as dropout and recruitment delays [25]. This enables the estimation of quantities of interest, such as the effect size to be expected, the proportion of the sample likely to experience an adverse effect, or the statistical power of the simulated trial [26]. Similar to JITAI-Twins, these models and simulations are designed meticulously, as the simulation results will be used to make decisions about the actual trial [27]. Unlike JITAI-Twins, clinical trial simulations are primarily used to optimize trial design or fix dosage regimens prior to the trial. JITAI-Twins are instead used to evaluate decision-making algorithms that will affect the intervention’s adaptation, rather than to the experimental design or some fixed dosing regimen [28].

Exceptions to the use of clinical trial simulations to optimize trial design include work on model-informed precision dosing (MIPD). MIPD typically entails the use of participant-specific models to inform dosing over the course of a pharmaceutical’s prescription to a patient [29]. This dosing can be dictated by a RL algorithm that adapts dosing according to some observations from the patient. However, the simulations typically performed are used to inform dosing for a specific individual, rather than to inform the decision-making algorithm used for an entire trial population [30]. Augustin et al. extended this idea in a way most similar to JITAI-Twins with their recent work on clinical trial simulations to evaluate candidate MIPD algorithms [31]. Augustin et al. use clinical trial simulations to show that a mechanistic model-based control approach outperforms deep RL and black box model-based control in individualizing warfarin dosing regimens. This is similar to how JITAI-Twins are used to decide between candidate decision-making algorithms for a JITAI. However, JITAI-Twins differ in two important ways. First and foremost, clinical trial simulations only account for unidirectional information transfer: the use of virtual models to make decisions that affect some physical system. The JITAI-Twin framework explicitly accounts for information transfer back from the physical to the virtual: JITAI-Twins must be constructed using prior deployment data and are updated with new data each time a deployment is complete. Clinical trial simulations can be constructed without real world data [32]. The idea of updating these simulation models after a trial is complete to improve future simulations has not been formally discussed [23], [33]. Second, JITAI-Twins are not restricted to the use of model-based control algorithms [34], [35], [36]. Model-free control algorithms can also be evaluated using JITAI-Twins.

**Simulation Test Beds for RL Algorithms**

The RL literature has long used simulation test beds to evaluate algorithms [37]. However simulation-based evaluation has typically been used to demonstrate one algorithm’s superiority over others without necessitating deployment as the end goal. [38], [39]. This is in contrast to simulation for JITAI-Twins (and clinical trial simulations, for that matter). For JITAI-Twins, virtual simulations are a means to inform design choices for subsequent deployment. This distinction is important because the digital twin must then be carefully designed – designing a JITAI-Twin and evaluating candidate RL algorithms in simulation is often a project in and of itself. If simulation testbeds are not carefully designed, virtual evaluations are unlikely to translate to the physical twin. This is especially critical for “one-way door” settings such as clinical trials [40]. Decisions cannot be changed once a trial is initiated. This is higher stakes compared to RL for games, advertising, and other “two-way door” settings [38].

One notable exception to this view of simulation-based evaluation as an end goal is the Sim2Real body of work [41]. In Sim2Real, a RL algorithm is used to train an agent in a simulation environment. This trained agent is then deployed to the real world with the knowledge gained during simulation. For some Sim2Real methods, the agent is forced to purely exploit in the real world, leveraging the exploration knowledge gained via simulation (i.e., learning stops after simulation in an “offline” manner on “batch” data) [41]. Other Sim2Real methods allow the agent to fine tune (i.e., continue to learn) for continued exploration and exploitation in the real world. Methods that view Sim2Real as a closed loop are closest in principle to JITAI-Twins. Chebotar et al. detail how data obtained from real-world deployment can then be used to update the simulation testbed [42]. The RL agent then continues to learn with the updated simulation testbed and is then deployed again to the real world; and the cycle continues [43], [44]. Note that this closed-loop Sim2Real literature in fact constitutes an instantiation of digital twins. However, closed-loop Sim2Real still differs from JITAI-Twins in important ways. First and foremost, JITAI-Twins are a decision support tool: JITAI-Twins help answer the question, “Based on these virtual simulation results, which RL algorithm should be the personalizer for the JITAI?” On the other hand, closed-loop Sim2Real is an enhanced training tool for RL algorithms: Sim2Real takes a specific personalizer (RL algorithm) and helps it perform better in the real world by leveraging a virtual environment prior to physical deployment. This is critical to understand from a modeling perspective. JITAI-Twins do not model specific individuals and their behaviors. Instead, JITAI-Twins model the behavior of a target subpopulation, using prior deployment data as a guide. Because specific individuals and characteristics are not modeled, a specific RL algorithm’s learning via the virtual twin may not be applicable to its deployment for the physical twin. This is desirable because individuals recruited for an upcoming deployment are typically sampled from the target subpopulation, and samples differ from previous deployments’ samples. Hence, JITAI-Twins are used to make decisions about which RL algorithm performs best for the entire subpopulation, rather than to train a specific algorithm to perform well for specific settings in the real world. Closed-loop Sim2Real is instead more fit for digital twins in individualized settings, where information transfer from virtual to physical can include a specific RL algorithm’s training. Sim2Real has been used in mobile health settings to inform deployed RL algorithms (without fine tuning) [45], but no bidirectional feedback was employed from deployment data back to the simulation environment (i.e., closed-loop Sim2Real), unlike the JITAI-Twin framework.

**S2. Primer on RL Algorithms and Models for Simulation in the Context of JITAIs**

In this section, we provide an overview of concepts in modeling and simulation for RL that are particularly relevant to JITAIs and JITAI-Twins. We discuss RL algorithms with specific examples from the JITAI literature to make the mathematics more concrete. We then detail how modeling and simulation are used in the context of RL.

**RL Algorithms**

RL algorithms are designed to optimize *decisions* made over some (possibly infinite) sequence of time points or *decision times*. These decisions are optimized with respect to some *outcome* of interest. The outcomes are often dependent on the specific *context* in which decisions are made. In mHealth settings, a *decision* could be whether a JITAI sends a reminder to take a walk at specific *decision times* throughout the day [34], such as after lunch. An *outcome* of interest could be the observed step count over the next 30 minutes, and this outcome is likely to depend on the *context* in which the reminder was delivered in, such as the weather outside or an individual’s current physiological stress [46]. In RL, *decisions* are referred to as *actions*, $A_{t}$, taken at *decision times* or timesteps $t\in\{1, 2, \ldots,T\}$. $T$ can be infinite and is the number of timesteps available (e.g., number of possible times a JITAI might send a reminder in a 10-week deployment). *Outcomes* can be mapped to *rewards*, $R_{t}$, and *context* is referred to as *state*, $S_{t}$. Note that outcomes in a clinical sense may not map to the rewards for a RL algorithm. The reward for a RL algorithm is what the RL algorithm needs to optimize for, but that may not necessarily just be the outcome of interest in the clinical sense. For example, a clinician may want a JITAI to optimize an individual’s step counts over the course of some time, but the RL algorithm may also need to be penalized (i.e., negative reward) when it annoys a user so much that they perform less physical activity in the long run [47]. In this walking example, the decision to send a reminder or not could be modeled as a binary action, $A_{t}\in\left\{ 0,1 \right\}$, the reward could be some function of how many times the JITAI has already sent a push notification and subsequent step counts over the next 30 minutes. State, $S_{t}\in\mathbb{R}^{p}$, could be of dimension $p$, comprising of predictions and measurements of contextual variables such as the weather, an individual’s heart rate variability, or their sleep quality the previous night.

The goal of a JITAI’s RL algorithm is to select actions that maximize the total reward over the course of the deployment. More precisely, the RL algorithm seeks to learn the sequence of actions $\left\{ A_{t} \right\}_{t=1}^{T}$ that maximizes the cumulative reward, $\sum_{t=1}^{T} R_{t}$. The core challenge in doing so is that the RL algorithm has to do so with only estimates of the true reward function, $r\left( s,a \right)=E\left[ R_{t}|S_{t}=s, A_{t}=a \right]$ and the state transition function, $T\left( s^{'}, s, a \right)=P\left( S_{t+1}=s^{'} \right|S_{t}=s, A_{t}=a)$. Here, $E\left[ \cdot\right]$ represents the expectation. The RL algorithm must navigate this challenge of attempting to select optimal actions $A_{t}$ at each state $S_{t}$ without perfect knowledge of the reward or state transition functions, only estimates, $r\left( s,a \right)$ and $T\left( s^{'}, s, a \right)$. The RL algorithm thus has two competing goals: the first is to balance *exploration* by taking actions $A_{t}$ that help the algorithm learn what reward and next state, $R_{t}, S_{t+1},$ to expect given a certain context, $S_{t}$ (i.e., learning better $r\left( s,a \right), T\left( s^{'}, s, a \right)$). The second goal is *exploitation* by utilizing what the algorithm has learned thus far with $r\left( s,a \right), T\left( s^{'}, s, a \right)$ to select actions $\left\{ A_{t} \right\}_{t=1}^{T}$ that maximize $\sum_{t=1}^{T} R_{t}$. How a RL algorithm balances this exploration versus exploitation tradeoff is just one example of the many design decisions that must be made prior to a JITAI’s deployment with a RL algorithm [38].

Two additional technical considerations are worth noting for the RL algorithms used in JITAIs thus far. The first involves update times [38]. Update times are times when the agent updates its models of the reward function and/or transition function, $r\left( s,a \right), T\left( s^{'}, s, a \right),$ based on the history of data observed thus far, $\left\{ S_{k}, A_{k}, R_{k} \right\}_{k=1}^{t-1}$. In practical settings, updates prior to each decision time may not be feasible or desirable. Update times could instead be when a mHealth system is charging overnight because internet connectivity and power consumption are of less concern. These update times are important to consider when constructing JITAI-Twins for simulation. Update and decision times must be modeled and simulated to produce results that are faithful to deployment. If updates are made more or less frequently, this could produce disparate results.

The second consideration involves *pooling*, or the use of other participants’ data to inform a JITAI’s decisions [48]. Pooling trades decreased personalization in exchange for faster learning [49]. Our notation thus far suggests that a RL algorithm learns $r\left( s,a \right), T\left( s^{'}, s, a \right),$ solely based on what it has observed of an individual’s data, $\left\{ S_{i,k}, A_{i,k}, R_{i,k} \right\}_{k=1}^{t}$, where $i\in\left\{ 1, 2, \ldots, N \right\}$ indexes the individual $i$ in a deployment of $N$ participants. However, if the algorithm leverages pooling to learn based on other participants’ data, action $A_{i,t}$ may be selected based on $\left\{ \left\{ S_{i,k}, A_{i,k}, R_{i,k} \right\}_{k=1}^{t-1} \right\}_{i=1}^{N}$, or the data observed until timestep $t-1$ for all participants. A subset of $\left\{ 1, 2, \ldots, N \right\}$ could also be used. If pooling is employed, factors such as recruitment rate are especially important to simulate in a JITAI-Twin, as the data available to each JITAI’s agent will differ depending on the participants recruited thus far.

**Models for Simulation in RL**

Simulation testbeds in RL are virtual environments that are characterized by virtual constructs or models of the “world” [50]. From the agent’s perspective, the agent infers its current state, it takes an action, observes the reward, and the cycle repeats. From the world’s perspective, the agent takes an action, this action has some effect on the world (e.g., the individual, their health state, their environment, etc.). This effect is mapped to some outcome. This outcome along with other factors that influence the RL algorithm’s decision are used to generate a reward for the RL algorithm’s decision. Note the distinction here between the simulation testbed’s generated outcome and a specific RL algorithm’s reward. The world then changes according to what state it was in previously and what action the RL algorithm took – and the cycle continues. This distinction between the world’s perspective and the algorithm’s perspective is important when designing digital twins for settings where the JITAI’s RL algorithm may not be privy to particular aspects of the world [37]. For example, a JITAI may not be designed to use data from a wrist-worn photoplethysmogram (PPG) sensor. Hence, the agent may not be privy to changes in heart rate. However, this does not mean that heart rate does not change from decision time to decision time. From the perspective of the world, the outcomes produced could be influenced by the individual’s current heart rate (e.g., the efficacy of a JITAI’s mindfulness intervention could be influenced by stress) [51]. Mathematically, state, $S$, is the multidimensional variable where the distinction between agent and the world is important. For the remainder of the paper, we denote $S^{w}$ as the state of the world and $S$, as before, as the state of the algorithm. $S$ will always be some lossy mapping of $S^{w}$ (i.e., the agent never observes the world exactly, just as a JITAI system never has perfect sensing and inference capabilities) [52].

A simulation testbed simulates the environment that a RL algorithm is deployed in. Once designed, a simulation testbed allows for an algorithm to interact with a virtual environment in a way that mimics the algorithm’s operation during deployment. From the RL algorithm’s perspective, once it selects an action, $A_{t}$ in state $S_{t}$, it will receive some reward in return, $R_{t}$. The algorithm will then infer that it is in state $S_{t+1}$. It will select action $A_{t+1}$ and observe reward $R_{t+1}$. From the algorithm’s perspective, this process is the same regardless of its deployment in the virtual world or in the real world. What goes on outside of the RL algorithm’s purview will differ. During deployment, the RL algorithm will inform the JITAI of which action to take, $A_{t},$ in state $S_{t}$. The JITAI will then interact with the physical twin, measure the outcome framed as a reward, $R_{t}$, and infer the next state $S_{t+1}$ from observations (e.g., sensor data, survey responses, etc.). This information on $R_{t}$ and $S_{t+1}$ will be digitally transferred back to the RL agent and the cycle will continue. From a RL agent’s perspective, this process is equivalent for both the virtual and physical twins. This equivalence from the RL agent’s perspective is important to understand because it implies that although a physical environment may be necessary to evaluate a JITAI system in its entirety (e.g., smartphone-based JITAI) [53], a physical environment is not necessary to simulate a RL algorithm’s operation.

For any simulation testbed in RL, two virtual constructs are fundamental: the construct that generates outcomes at each timestep (that can be mapped to rewards for a particular RL algorithm) and the construct that generates the next timestep’s world state (that can be mapped to the RL algorithm’s inferred state). We refer to these constructs as the world’s outcome model and the world’s state transition model. The outcome model dictates what outcome will be produced, $R_{t}$, given the RL algorithm took action $A_{t}$ in state $S_{t}^{w}$. The model can be expressed stochastically as the probability mapping $y\left( s, a \right)=P\left( Y_{t}=y | S_{t}^{w}=s, A_{t}=a \right)$. This outcome model is a multidimensional probability density function, where for each combination of a RL algorithm’s actions and the world’s states, the possible outcomes are associated with some probability density. The world’s state transition model dictates what state the environment should proceed to next given the RL algorithm’s action and the world’s current state, $W\left( s', s, a \right)=P\left( S_{t+1}^{w}=s^{'} \right|S_{t}^{w}=s, A_{t}=a)$. This mapping is also probabilistic and produces a multidimensional probability density function, where each combination of world state and agent action is then mapped to all possible next states with associated probabilities.

Two additional models necessary for a specific RL algorithm’s evaluation are the mapping from $S^{w}\to S$ (i.e., what aspects of the world’s state will the algorithm be privy to) and the mapping from $Y\to R$ (i.e., what aspects of the world’s outcomes does the algorithm use to reward its decision making). These two world-to-algorithm mappings are specified based on the upcoming JITAI system design and trial. If particular aspects of the world (i.e., elements of $S^{w}$) are important to the outcomes of a RL algorithm’s actions – but these aspects are not inferred or measurable by the RL algorithm – then the RL algorithm’s state, $S$, will include a subset of imperfect information from $S^{w}$. This is important to model virtually because from the RL algorithm’s perspective, it will attempt to learn $r\left( s,a \right)= E\left[ R_{t}=r \right|S_{t}=s, A_{t}=a$] and not $y\left( s, a \right)=P\left( Y_{t}=y | S_{t}^{w}=s, A_{t}=a \right)$. As is clear from the notation, this learning will have access to $S_{t}$ – not $S_{t}^{w}$.

In practice, generative reward and state transition models can be used to construct simulation environments of varying complexity. For a robot traversing some difficult terrain, the world’s state can encompass high-resolution information on aspects of the terrain, the weather, etc. The agent’s state can be pixel-by-pixel representations of what the robot will be able to see via video when deployed in the difficult terrain. Similar high-resolution examples can be provided for video games or self-driving cars [54]. On the other hand, a simulation environment can be much less visually detailed if the RL algorithm does not expect to have visual capabilities. For JITAIs, video data are rarely available to mHealth systems (in contrast with telehealth systems) [55]. Hence, the simulation environment does not need to reconstruct the physical environment that an individual will operate in. Rather, only the variables necessary to understand the effects of RL algorithm actions on the reward are necessary to model with fidelity. For example, rather than modeling the world as a visual scene of an individual walking around on a sunny day with pixel-by-pixel details, the world model can simply consist of a set of weather variables, a global positioning system variable for the individual’s location, and other summary factors that mediate the relationship between walking reminders and the individual’s subsequent step count, along with other factors affecting the reward (e.g., user burden associated with sending a walking suggestion). Thus, JITAI-Twins as described in this paper will be elucidated via the mathematical models used to generate outcomes and next states when the RL agent takes actions. This guidance is programming language-agnostic and does not require any sophisticated aesthetics. The variables can be visualized as columns in a table.

**S3. Additional Examples of Data Impoverishment Addressed in Prior Work**

For HeartSteps V2, variables relevant to an individual’s context or state were first impoverished [34]. The HeartSteps V1 MRT did not include measures of app engagement [56]. However, the study team learned during the MRT that app engagement would be important to consider when delivering interventions in the subsequent JITAI trial. To overcome this impoverishment in a state variable, the binary app engagement variable in the virtual twin had to be generated. Sampling bias was another data-deployment mismatch that existed. Within the subpopulation of sedentary adults, the HeartSteps MRT was conducted in sedentary adults who were not diagnosed with any cardiovascular disease. However, the JITAI was to be deployed in a hypertensive sample. This sampling bias was acknowledged at the time but was determined to be less consequential to the JITAI-Twin’s simulation results because sedentary behavior itself (rather than sedentary behavior’s effects on other health outcomes) was assumed to be similar [57].

We provide one additional example of data impoverishment to outline how prior work has addressed impoverishment in an intervention (i.e., action) variable. For our team’s work on a JITAI for oral health, no interventions were delivered in the prior data – in other words, the action variable was impoverished [49]. Responsiveness to the proposed intervention had to be imputed to enable reward generation for the JITAI-Twin’s simulations. Imputation was done by drawing user-specific treatment effect sizes deemed reasonable by domain scientists from zero-truncated normal distributions. Zero truncation assumed that the minimum level of responsiveness to an intervention would be zero. In other words, a mHealth reminder would not have a negative effect on the likelihood of brushing or the time spent brushing. Note that the delayed negative effects expected of a mHealth intervention on user engagement were also modeled for the RL algorithm’s reward function. The zero-truncated normal distributions’ means and variances were informed by parameters learned from prior data. The distributions characterized the plausible effect sizes for how likely an intervention would affect the probability of brushing (modeled as a Bernoulli probability) and how much the intervention would increase brushing time (Poisson distribution). Note that when estimating the effects of actions and intervention options, it is important for the team to realistically approximate the range of effect sizes possible. Digital therapeutics tend to have a relatively small exogenous impact on state dynamics, relative to the state’s autonomous dynamics and residual noise. Thus, the generated rewards must account for these small effect sizes [58].

The three examples we provide (including the HeartSteps V3 example included in the main text) summarize our approaches to handle data impoverishment in state, action, and outcome variables. The key intuition behind each approach is to impute variables in scientifically plausible ways, informed by variables and statistics from the available data. If state elements are impoverished, these variables can be generated with information on how these states evolve in practice. The statistics of available state elements can be used to inform the probability distributions for the impoverished state elements. If rewards are impoverished, a simulated reward signal can be generated using information from alternative outcomes, mediators, and effect sizes available and learned from prior data. If actions are impoverished, effect sizes can be imputed by consulting the relevant domain science and leveraging other aspects of the data to inform the distributions used in sampling effect sizes for each simulated trial participant.

**S4. Open Questions and Future Directions in JITAI-Twin Design**

JITAIs are nascent and mHealth technology is advancing rapidly [59], [60]. This necessitates continual updating of JITAI-Twins using present methods, as discussed thus far. However, this growth also presents an opportunity to improve methods for JITAI-Twin design and evaluation. The remainder of this section outlines open questions and future directions for JITAI-Twin methods development.

How can we leverage calibration when constructing simulation testbed variants? A challenge faced when constructing variants of a simulation testbed is that matching conditions must be met when varying the parameters under investigation. These matching conditions or marginal statistics typically involve characteristics of the trial, study population, or intervention that are known and should not vary in simulation. However, varying parameters under investigation may indirectly impact these statistics – for example, the standardized average treatment effect of a digital therapeutic [61]. Currently, variants are designed by varying the parameters under investigation within plausible ranges. A grid search is performed, and only the variants that retain the desired matching conditions are used for simulation. This is inefficient and should be improved upon. The rich model calibration literature may be a source of guidance in this regard [62].

Can foundation models be used to accelerate JITAI-Twin design? Deployment subpopulations are affected by similar phenomena across JITAI studies. Examples include habituation to repeated delivery of the intervention, user burden in response to an overwhelming number of interactions, and increasing disengagement with the intervention over time. The lack of prior deployment data available for any particular application often forces the use of JITAI-Twin models with few parameters. If more flexible models were instead trained on big data and then fine-tuned to particular applications, trial population behavior could potentially be modeled more accurately. This is analogous to foundation transformer models (e.g., BERT) being fine-tuned to specific applications (e.g., medBERT) [63]. Participant privacy may preclude the near-term curation of a large corpus of JITAI deployment data in clinical trial populations. However, an opportunity may exist in leveraging the wealth of wellness data collected by commercial wearables and smartphones. Wearables and mHealth apps available on app stores face similar challenges of disengagement, user burden, and habituation. The volume of data available may outweigh the lack of control and noisiness of the data [64]. Future work is necessary to evaluate the tradeoffs of training and fine-tuning foundation models for JITAI-Twins.

What additional steps can be taken to improve JITAI-Twin evaluation before making JITAI-Twin-informed decisions for an upcoming deployment? JITAI-Twin evaluation involves verification, validation, and uncertainty quantification (VVUQ), as discussed. Verification methods are mature, but validation and uncertainty quantification methods for JITAI-Twins could use further development. Current validation approaches suffer from “the chicken or the egg” problem. A JITAI-Twin’s simulations cannot truly be validated without data from the target deployment. However, once the target deployment has taken place, validating the prior version of the JITAI-Twin is no longer useful. Is this problem fundamental to JITAI-Twins, or are there alternative ways that a JITAI-Twins can be validated and used for improved decision-making prior to the target deployment? From an uncertainty quantification perspective, JITAI-Twin research has focused primarily on sensitivity analyses, constructing simulation testbed variants, and forming confidence intervals over several simulated deployments. However, Bayesian uncertainty propagation could also be applied directly to the generative models themselves to inform simulation outcomes [65]. This uncertainty quantification could provide a more comprehensive picture of simulation results and better inform algorithm decisions.

**References**

[1] M. W. Grieves, “Digital Twins: Past, Present, and Future,” *The Digital Twin*, vol. 1, pp. 97–121, Jan. 2023, doi: 10.1007/978-3-031-21343-4_4/FIGURES/8.

[2] E. H. Glaessgen and D. S. Stargel, “The Digital Twin Paradigm for Future NASA and U.S. Air Force Vehicles,” 2012.

[3] C. F. Elliott, J. Duncan, T. M. Tang, M. Behr, K. Kumbier, and B. Yu, “Designing a Data Science simulation with MERITS: A Primer,” Mar. 2024, Accessed: Aug. 13, 2024. [Online]. Available: https://arxiv.org/abs/2403.08971v1

[4] L. P. Kaelbling, M. L. Littman, and A. W. Moore, “Reinforcement Learning:  A Survey,” *Journal of Artificial Intelligence Research*, vol. 4, pp. 237–285, May 1996, doi: 10.1613/JAIR.301.

[5] P. I. Watts *et al.*, “Healthcare Simulation Standards of Best PracticeTM Simulation Design,” *Clin Simul Nurs*, vol. 58, pp. 14–21, Sep. 2021, doi: 10.1016/j.ecns.2021.08.009.

[6] National Academies of Sciences Engineering and Medicine, “Foundational Research Gaps and Future Directions for Digital Twins,” *Washington, DC: The National Academies Press*, pp. 1–202, 2024, doi: 10.17226/26894.

[7] National Academies of Sciences Engineering and Medicine, “Opportunities and Challenges for Digital Twins in Biomedical Research: Proceedings of a Workshop in Brief (2023),” *National Academies Press (US)*, pp. 1–13, 2023, doi: 10.17226/26922.

[8] L. Li, S. Aslam, A. Wileman, and S. Perinpanayagam, “Digital Twin in Aerospace Industry: A Gentle Introduction,” *IEEE Access*, vol. 10, pp. 9543–9562, 2022, doi: 10.1109/ACCESS.2021.3136458.

[9] F. Tao, H. Zhang, A. Liu, and A. Y. C. Nee, “Digital Twin in Industry: State-of-the-Art,” *IEEE Trans Industr Inform*, vol. 15, no. 4, pp. 2405–2415, Apr. 2019, doi: 10.1109/TII.2018.2873186.

[10] M. F. Bolus, A. A. Willats, C. J. Rozell, and G. B. Stanley, “State-space optimal feedback control of optogenetically driven neural activity,” *J Neural Eng*, vol. 18, no. 3, p. 036006, Mar. 2021, doi: 10.1088/1741-2552/ABB89C.

[11] E. Katsoulakis *et al.*, “Digital twins for health: a scoping review,” *NPJ Digit Med*, vol. 7, no. 1, pp. 1–11, Mar. 2024, doi: 10.1038/s41746-024-01073-0.

[12] M. Di Shen, S. B. Chen, and X. D. Ding, “The effectiveness of digital twins in promoting precision health across the entire population: a systematic review,” *npj Digital Medicine 2024 7:1*, vol. 7, no. 1, pp. 1–10, Jun. 2024, doi: 10.1038/s41746-024-01146-0.

[13] K. P. Venkatesh, G. Brito, and M. N. Kamel Boulos, “Health Digital Twins in Life Science and Health Care Innovation,” *Annu Rev Pharmacol Toxicol*, vol. 64, no. Volume 64, 2024, pp. 159–170, Jan. 2024, doi: 10.1146/ANNUREV-PHARMTOX-022123-022046/CITE/REFWORKS.

[14] K. Zhang *et al.*, “Concepts and applications of digital twins in healthcare and medicine,” *Patterns*, vol. 5, no. 8, p. 101028, Aug. 2024, doi: 10.1016/J.PATTER.2024.101028.

[15] A. Vallée, “Envisioning the Future of Personalized Medicine: Role and Realities of Digital Twins,” *J Med Internet Res 2024;26:e50204 https://www.jmir.org/2024/1/e50204*, vol. 26, no. 1, p. e50204, May 2024, doi: 10.2196/50204.

[16] M. De Domenico *et al.*, “Challenges and opportunities for digital twins in precision medicine from a complex systems perspective,” *npj Digital Medicine 2025 8:1*, vol. 8, no. 1, pp. 1–11, Jan. 2025, doi: 10.1038/s41746-024-01402-3.

[17] S. Elkefi and O. Asan, “Digital Twins for Managing Health Care Systems: Rapid Literature Review,” *J Med Internet Res*, vol. 24, no. 8, Aug. 2022, doi: 10.2196/37641.

[18] M. N. Kamel Boulos and P. Zhang, “Digital Twins: From Personalised Medicine to Precision Public Health,” *Journal of Personalized Medicine 2021, Vol. 11, Page 745*, vol. 11, no. 8, p. 745, Jul. 2021, doi: 10.3390/JPM11080745.

[19] L. Kolla, F. K. Gruber, O. Khalid, C. Hill, and R. B. Parikh, “The Case for AI-Driven Cancer Clinical Trials – The Efficacy Arm In Silico,” *Biochim Biophys Acta Rev Cancer*, vol. 1876, no. 1, p. 188572, Aug. 2021, doi: 10.1016/J.BBCAN.2021.188572.

[20] E. Katsoulakis *et al.*, “Digital twins for health: a scoping review,” *NPJ Digit Med*, vol. 7, no. 1, pp. 1–11, Mar. 2024, doi: 10.1038/s41746-024-01073-0.

[21] P. M. Thangaraj, S. V. Shankar, E. K. Oikonomou, and R. Khera, “RCT-Twin-GAN Generates Digital Twins of Randomized Control Trials Adapted to Real-world Patients to Enhance their Inference and Application,” *medRxiv*, Dec. 2023, doi: 10.1101/2023.12.06.23299464.

[22] P. Armeni, I. Polat, L. M. De Rossi, L. Diaferia, S. Meregalli, and A. Gatti, “Digital Twins in Healthcare: Is It the Beginning of a New Era of Evidence-Based Medicine? A Critical Review,” *J Pers Med*, vol. 12, no. 8, Aug. 2022, doi: 10.3390/JPM12081255.

[23] N. Holford, S. C. Ma, and B. A. Ploeger, “Clinical trial simulation: a review,” *Clin Pharmacol Ther*, vol. 88, no. 2, pp. 166–182, Aug. 2010, doi: 10.1038/CLPT.2010.114.

[24] H. C. . Kimko and S. B. . Duffull, “Simulation for designing clinical trials : a pharmacokinetic-pharmacodynamic modeling perspective,” p. 396, 2003.

[25] P. Girard, “Clinical Trial Simulation: A Tool for Understanding Study Failures and Preventing Them,” *Basic Clin Pharmacol Toxicol*, vol. 96, no. 3, pp. 228–234, Mar. 2005, doi: 10.1111/J.1742-7843.2005.PTO960313.X.

[26] Z. Chen *et al.*, “Exploring the feasibility of using real-world data from a large clinical data research network to simulate clinical trials of Alzheimer’s disease,” *npj Digital Medicine 2021 4:1*, vol. 4, no. 1, pp. 1–9, May 2021, doi: 10.1038/s41746-021-00452-1.

[27] N. H. Holford *et al.*, “Simulation in drug development: good practices,” 1999. Accessed: Feb. 23, 2025. [Online]. Available: https://holford.fmhs.auckland.ac.nz/docs/simulation-in-drug-development-good-practices.pdf

[28] P. L. Bonate, “Clinical trial simulation in drug development,” *Pharm Res*, vol. 17, no. 3, pp. 252–256, 2000, doi: 10.1023/A:1007548719885/METRICS.

[29] A. S. Darwich *et al.*, “Model-Informed Precision Dosing: Background, Requirements, Validation, Implementation, and Forward Trajectory of Individualizing Drug Therapy,” *Annu Rev Pharmacol Toxicol*, vol. 61, no. 1, pp. 361–3621, 2021, doi: 10.1146/ANNUREV-PHARMTOX-033020-113257.

[30] J. S. Pérez-Blanco and J. M. Lanao, “Model-Informed Precision Dosing (MIPD),” *Pharmaceutics 2022, Vol. 14, Page 2731*, vol. 14, no. 12, p. 2731, Dec. 2022, doi: 10.3390/PHARMACEUTICS14122731.

[31] D. Augustin, B. Lambert, M. Robinson, K. Wang, and D. Gavaghan, “Simulating clinical trials for model-informed precision dosing: using warfarin treatment as a use case,” *Front Pharmacol*, vol. 14, p. 1270443, Oct. 2023, doi: 10.3389/FPHAR.2023.1270443.

[32] H. Kimko and K. Lee, “Improving Realism in Clinical Trial Simulations via Real‐World Data,” *CPT Pharmacometrics Syst Pharmacol*, vol. 6, no. 11, p. 727, Nov. 2017, doi: 10.1002/PSP4.12232.

[33] M. Viceconti, A. Henney, and E. Morley-Fletcher, “In silico clinical trials: how computer simulation will transform the biomedical industry,” *Int J Clin Trials*, vol. 3, no. 2, pp. 37–46, May 2016, doi: 10.18203/2349-3259.IJCT20161408.

[34] P. Liao, K. Greenewald, P. Klasnja, and S. Murphy, “Personalized HeartSteps: A Reinforcement Learning Algorithm for Optimizing Physical Activity,” *Proc ACM Interact Mob Wearable Ubiquitous Technol*, vol. 4, no. 1, Mar. 2020, doi: 10.1145/3381007.

[35] S. Ghosh *et al.*, “MiWaves Reinforcement Learning Algorithm,” Aug. 2024, Accessed: Sep. 11, 2024. [Online]. Available: https://arxiv.org/abs/2408.15076v1

[36] A. L. Trella *et al.*, “A Deployed Online Reinforcement Learning Algorithm In An Oral Health Clinical Trial,” Sep. 2024, Accessed: Sep. 11, 2024. [Online]. Available: https://arxiv.org/abs/2409.02069v1

[37] R. Sutton and A. Barto, *Reinforcement learning: An introduction*. 2018.

[38] A. L. Trella, K. W. Zhang, I. Nahum-Shani, V. Shetty, F. Doshi-Velez, and S. A. Murphy, “Designing Reinforcement Learning Algorithms for Digital Interventions: Pre-Implementation Guidelines,” *Algorithms*, vol. 15, no. 8, p. 255, Jul. 2022, doi: 10.3390/A15080255.

[39] G. Dulac-Arnold *et al.*, “Challenges of real-world reinforcement learning: definitions, benchmarks and analysis,” *Mach Learn*, vol. 110, no. 9, pp. 2419–2468, Sep. 2021, doi: 10.1007/S10994-021-05961-4/FIGURES/11.

[40] J. P. Bezos, “1997 Letter to Amazon Shareholders.”

[41] W. Zhao, J. P. Queralta, and T. Westerlund, “Sim-to-Real Transfer in Deep Reinforcement Learning for Robotics: A Survey,” *2020 IEEE Symposium Series on Computational Intelligence, SSCI 2020*, pp. 737–744, Dec. 2020, doi: 10.1109/SSCI47803.2020.9308468.

[42] Y. Chebotar *et al.*, “Closing the Sim-to-Real Loop: Adapting Simulation Randomization with Real World Experience,” *Proc IEEE Int Conf Robot Autom*, vol. 2019-May, pp. 8973–8979, Oct. 2018, doi: 10.1109/ICRA.2019.8793789.

[43] S. Abeyruwan *et al.*, “i-Sim2Real: Reinforcement Learning of Robotic Policies in Tight Human-Robot Interaction Loops,” *Proc Mach Learn Res*, vol. 205, pp. 212–224, Jul. 2022, Accessed: Aug. 13, 2024. [Online]. Available: https://arxiv.org/abs/2207.06572v4

[44] A. Farchy, S. Barrett, P. Macalpine, and P. Stone, “Humanoid Robots Learning to Walk Faster: From the Real World to Simulation and Back,” 2013. Accessed: Aug. 13, 2024. [Online]. Available: www.ifaamas.org

[45] E. Tragos *et al.*, “Keeping People Active and Healthy at Home Using a Reinforcement Learning-based Fitness Recommendation Framework,” *IJCAI International Joint Conference on Artificial Intelligence*, vol. 2023-August, pp. 6237–6245, 2023, doi: 10.24963/IJCAI.2023/692.

[46] S. L. Battalio *et al.*, “Sense2Stop: A micro-randomized trial using wearable sensors to optimize a just-in-time-adaptive stress management intervention for smoking relapse prevention,” *Contemp Clin Trials*, vol. 109, Oct. 2021, doi: 10.1016/J.CCT.2021.106534.

[47] P. LIAO *et al.*, “Just-in-Time but Not Too Much: Determining Treatment Timing in Mobile Health,” *Proc ACM Interact Mob Wearable Ubiquitous Technol*, vol. 2, no. 4, pp. 1–21, Dec. 2018, doi: 10.1145/3287057.

[48] S. Tomkins, P. Liao, P. Klasnja, and S. Murphy, “IntelligentPooling: Practical Thompson Sampling for mHealth,” *Mach Learn*, vol. 110, no. 9, pp. 2685–2727, Jul. 2020, doi: 10.1007/s10994-021-05995-8.

[49] A. L. Trella, K. W. Zhang, I. Nahum-Shani, V. Shetty, F. Doshi-Velez, and S. A. Murphy, “Reward Design for an Online Reinforcement Learning Algorithm Supporting Oral Self-Care,” *Proceedings of the AAAI Conference on Artificial Intelligence*, vol. 37, no. 13, pp. 15724–15730, Sep. 2023, doi: 10.1609/AAAI.V37I13.26866.

[50] D. Ha and J. Schmidhuber, “World Models,” *Forecasting in Business and Economics*, pp. 201–209, Mar. 2018, doi: 10.5281/zenodo.1207631.

[51] C. M. Raio, T. A. Orederu, L. Palazzolo, A. A. Shurick, and E. A. Phelps, “Cognitive emotion regulation fails the stress test,” *Proc Natl Acad Sci U S A*, vol. 110, no. 37, pp. 15139–15144, Sep. 2013, doi: 10.1073/PNAS.1305706110.

[52] I. Chadès, L. V. Pascal, S. Nicol, C. S. Fletcher, and J. Ferrer-Mestres, “A primer on partially observable Markov decision processes (POMDPs),” *Methods Ecol Evol*, vol. 12, no. 11, pp. 2058–2072, Nov. 2021, doi: 10.1111/2041-210X.13692.

[53] W. Hardeman, J. Houghton, K. Lane, A. Jones, and F. Naughton, “A systematic review of just-in-time adaptive interventions (JITAIs) to promote physical activity,” *International Journal of Behavioral Nutrition and Physical Activity*, vol. 16, no. 1, Apr. 2019, doi: 10.1186/S12966-019-0792-7.

[54] Y. Li, “Deep Reinforcement Learning: An Overview,” Jan. 2017, Accessed: Aug. 13, 2024. [Online]. Available: https://arxiv.org/abs/1701.07274v6

[55] A. R. Taha *et al.*, “The integration of mHealth technologies in telemedicine during the COVID-19 era: A cross-sectional study,” *PLoS One*, vol. 17, no. 2, Feb. 2022, doi: 10.1371/JOURNAL.PONE.0264436.

[56] P. Klasnja *et al.*, “Efficacy of Contextually Tailored Suggestions for Physical Activity: A Micro-randomized Optimization Trial of HeartSteps,” *Annals of Behavioral Medicine*, vol. 53, no. 6, pp. 573–582, May 2019, doi: 10.1093/ABM/KAY067.

[57] P. Liao, K. Greenewald, P. Klasnja, and S. Murphy, “Personalized HeartSteps: A Reinforcement Learning Algorithm for Optimizing Physical Activity,” *Proc ACM Interact Mob Wearable Ubiquitous Technol*, vol. 4, no. 1, Mar. 2020, doi: 10.1145/3381007.

[58] B. Luers, P. Klasnja, and S. Murphy, “Standardized Effect Sizes for Preventive Mobile Health Interventions in Micro-randomized Trials,” *Prevention Science*, vol. 20, no. 1, pp. 100–109, Jan. 2019, doi: 10.1007/S11121-017-0862-5/FIGURES/4.

[59] I. Nahum-Shani *et al.*, “Just-in-time adaptive interventions (JITAIs) in mobile health: Key components and design principles for ongoing health behavior support,” *Annals of Behavioral Medicine*, vol. 52, no. 6, pp. 446–462, May 2018, doi: 10.1007/S12160-016-9830-8/TABLES/2.

[60] J. McCool, R. Dobson, R. Whittaker, and C. Paton, “Mobile Health (mHealth) in Low- and Middle-Income Countries,” *Annu Rev Public Health*, vol. 43, no. Volume 43, 2022, pp. 525–539, Apr. 2022, doi: 10.1146/ANNUREV-PUBLHEALTH-052620-093850/CITE/REFWORKS.

[61] S. F. Anderson, “Appropriately estimating the standardized average treatment effect with missing data: A simulation and primer,” *Behav Res Methods*, vol. 56, no. 1, pp. 199–232, Jan. 2024, doi: 10.3758/S13428-022-02043-8/FIGURES/4.

[62] D. Bingham, T. Butler, and D. Estep, “Inverse Problems for Physics-Based Process Models,” *Annu Rev Stat Appl*, vol. 11, no. 1, pp. 461–482, Apr. 2024, doi: 10.1146/ANNUREV-STATISTICS-031017-100108.

[63] L. Rasmy, Y. Xiang, Z. Xie, C. Tao, and D. Zhi, “Med-BERT: pretrained contextualized embeddings on large-scale structured electronic health records for disease prediction,” *npj Digital Medicine 2021 4:1*, vol. 4, no. 1, pp. 1–13, May 2021, doi: 10.1038/s41746-021-00455-y.

[64] A. Veit, N. Alldrin, G. Chechik, I. Krasin, A. Gupta, and S. Belongie, “Learning From Noisy Large-Scale Datasets With Minimal Supervision,” *Proceedings - 30th IEEE Conference on Computer Vision and Pattern Recognition, CVPR 2017*, vol. 2017-January, pp. 6575–6583, Jan. 2017, doi: 10.1109/CVPR.2017.696.

[65] R. van de Schoot *et al.*, “Bayesian statistics and modelling,” *Nature Reviews Methods Primers 2021 1:1*, vol. 1, no. 1, pp. 1–26, Jan. 2021, doi: 10.1038/S43586-020-00001-2.
